# Fetal articular cartilage regeneration versus adult fibrocartilaginous repair: secretome proteomics unravels molecular mechanisms in an ovine model

**DOI:** 10.1242/dmm.033092

**Published:** 2018-07-06

**Authors:** Iris Ribitsch, Rupert L. Mayer, Monika Egerbacher, Simone Gabner, Maciej M. Kańduła, Julie Rosser, Eva Haltmayer, Ulrike Auer, Sinan Gültekin, Johann Huber, Andrea Bileck, David P. Kreil, Christopher Gerner, Florien Jenner

**Affiliations:** 1 VETERM, University Equine Hospital, University of Veterinary Medicine Vienna, Vienna 1210, Austria; 2 Department of Analytical Chemistry, Faculty of Chemistry, University of Vienna, Vienna 1090, Austria; 3 Histology & Embryology, Department of Pathobiology, University of Veterinary Medicine Vienna, Vienna 1210, Austria; 4 Department of Biotechnology, Boku University Vienna, Vienna 1180, Austria; 5 Institute of Bioinformatics, Johannes Kepler University, Linz 4040, Austria; 6 Department of Companion Animals and Horses, University of Veterinary Medicine Vienna, Vienna 1210, Austria; 7 Teaching and Research Farm Kremesberg, Clinical Unit for Herd Health Management in Ruminants, Department for Farm Animals and Veterinary Public Health, University of Veterinary Medicine Vienna, Vienna 1210, Austria

**Keywords:** Articular cartilage, Osteoarthritis, Regeneration, Proteome, Fetus

## Abstract

Osteoarthritis (OA), a degenerative joint disease characterized by progressive cartilage degeneration, is one of the leading causes of disability worldwide owing to the limited regenerative capacity of adult articular cartilage. Currently, there are no disease-modifying pharmacological or surgical therapies for OA. Fetal mammals, in contrast to adults, are capable of regenerating injured cartilage in the first two trimesters of gestation. A deeper understanding of the properties intrinsic to the response of fetal tissue to injury would allow us to modulate the way in which adult tissue responds to injury. In this study, we employed secretome proteomics to compare fetal and adult protein regulation in response to cartilage injury using an ovine cartilage defect model. The most relevant events comprised proteins associated with the immune response and inflammation, proteins specific for cartilage tissue and cartilage development, and proteins involved in cell growth and proliferation. Alarmins S100A8, S100A9 and S100A12 and coiled-coil domain containing 88A (CCDC88A), which are associated with inflammatory processes, were found to be significantly upregulated following injury in adult, but not in fetal animals. By contrast, cartilage-specific proteins like proteoglycan 4 were upregulated in response to injury only in fetal sheep postinjury. Our results demonstrate the power and relevance of the ovine fetal cartilage regeneration model presented here for the first time. The identification of previously unrecognized modulatory proteins that plausibly affect the healing process holds great promise for potential therapeutic interventions.

## Abstract (precis)

**Summary:** Secretome proteomics identifies differential regulation of inflammation modulators during fetal and adult articular cartilage defect healing, offering novel strategies for therapy.

## INTRODUCTION

Osteoarthritis (OA), a degenerative joint disease characterized by progressive articular cartilage degeneration, is one of the most commonly diagnosed diseases in general practice and one of the leading causes of disability worldwide (Barbour et al., 2013; Johnson and Hunter, 2014; Lopez and Murray, 1998; Woolf and Pfleger, 2003). In addition to its significant medical, social and psychological impact on quality of life, OA is associated with commensurate socioeconomic costs (Rabenda et al., 2007; Yelin et al., 2007). As adult articular cartilage has little intrinsic repair capacity and current treatment options are mostly palliative, the disease prevalence and burden places a strong emphasis on the need for new therapeutic strategies that could modify the structural progression of the disease and regenerate articular cartilage. The development of disease-modifying anti-OA drugs has thus far proven to be challenging, owing to the complexity of the disease and the pathophysiological pathways that drive OA progression. OA has a multifactorial aetiopathogenesis involving genetic, molecular and biomechanical influences, as well as lifestyle and environmental stress stimuli. Nevertheless, OA culminates in a consistent molecular, structural and clinical sequence of disease progression, characterized by inflammation, gradual loss of proteoglycans, collagen type II (COL2) degradation, cartilage fibrillation, loss of maturational arrest and phenotypic stability of articular chondrocytes, as reviewed previously (Goldring et al., 2011; Pap and Korb-Pap, 2015).

Fetal mammals, by contrast to adults, are capable of regenerating injured tissues including skin, palate, tendon, bone and cartilage in the first two trimesters of gestation (Al-Qattan et al., 1993; Degen and Gourdie, 2012; Kumahashi et al., 2004; Longaker et al., 1992, 1990; Namba et al., 1998; Stone, 2000; Wagner et al., 2001; Walker et al., 2000). Despite progress over the past decade, the mechanisms of scarless fetal healing and the embryogenetic mechanisms of articular chondrogenesis remain largely unknown (Decker et al., 2017; Jenner et al., 2014a,b; Lo et al., 2012).

Hence, in this study, we had three key aims: (1) to establish a standardized cartilage lesion model allowing comparison of cartilage healing in adult and fetal sheep (*Ovis* *aries*); (2) to establish the feasibility, repeatability and relevance of proteomic analysis of minute fetal and adult cartilage samples; and (3) to compare fetal and adult protein regulation in response to cartilage injury.

The proteomic analysis of the differential response of fetal and adult cartilage to injury will make a major contribution to our understanding of cartilage biology and help us to elucidate the molecular mechanisms underlying OA and cartilage regeneration. Such insights could help us to identify and target factors that are crucial to promote a regenerative response and might allow the development of disease-modifying treatment strategies to induce cartilage regeneration in adult mammals. A major challenge for the proteomic characterization of the complex protein mixture in cartilage extract is the wide dynamic range of protein abundance, making the detection of low-abundance proteins very difficult (Stenberg et al., 2013; Wilson and Bateman, 2008). Although technically demanding, however, studying the functional proteome gives a more comprehensive picture of disease aetiopathogenesis than gene expression analysis alone, as its interpretation is not limited by a possible disparity between gene and protein expression levels (Ritter et al., 2013)*.*

## RESULTS

### The ovine model supports complex surgical manipulations required for the investigation of cartilage regeneration

**Fig. 1.**
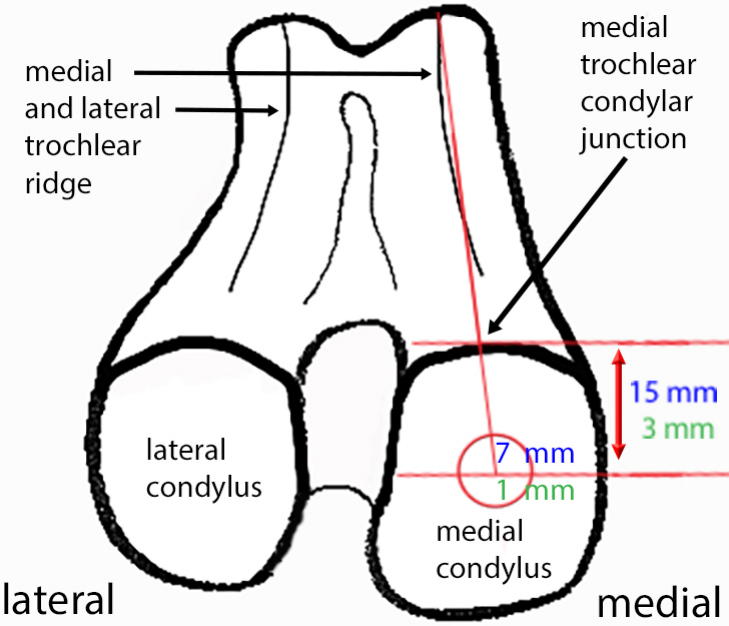
**Diagram of the distal femur.** The medial and lateral trochlea ridge and the medial and lateral condyle are identified as landmarks. Cartilage lesion location and size is indicated in blue in adult and green in fetal sheep. The lesion was centred 15 mm (adult) and 3 mm (fetus) distant from the medial trochlear-condylar junction on a line that virtually extended the medial trochlear ridge.

Ewes and fetal sheep tolerated the laparotomy, uterotomy and fetal manipulation well. No postoperative complications or abortions were encountered. Fetal sheep at 80 days of gestation (gd) had age-appropriate crown-anus lengths within the reported range of 10.1±1.3 cm (Mufti et al., 2000). The landmarks for standardized induction of medial femoral condylar cartilage lesions (Fig. 1) were easily identified and allowed placement of the lesion in the centre of the condyle.

### Long-term evaluation confirmed fetal regenerative versus adult scarring cartilage repair

In the pilot study designed as a proof-of-principle of fetal regeneration at 28 days postoperatively versus adult scarring repair at 5 months postinjury, the defect was macroscopically undetectable in fetal sheep resulting in an OARSI (Osteoarthritis Research Society International) macroscopic score (Little et al., 2010) of 0 for cartilage, osteophytes and synovium. By contrast, in adult sheep the defect was clearly evident and only partially filled with fibrocartilaginous tissue resulting in an OARSI macroscopic score of 5/16 for cartilage (surface roughening plus defect), 0/5 for osteophytes and 2/5 for synovium.

**Fig. 2.**
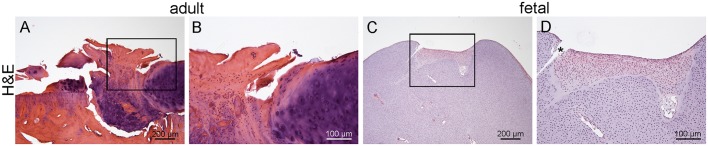
**Healing of adult (5 months postinjury) and fetal (28 days postinjury) cartilage defects: H&E-stained sections.** (A,B) In adult sheep no repair is apparent except in areas of microfracture. Only minimal fibrocartilage is evident with partial hyalinization; there is no integration with surrounding cartilage (see insert). (C,D) In fetal sheep the defect is 80-90% filled with differentiating hyaline cartilage and the superficial 10-20% with repair tissue with progressing hyalinization; there is good integration with surrounding cartilage. *Processing artifact.

Histologically (Fig. 2), no defect repair and only minimal fibrocartilaginous regeneration adjacent to microfractures without integration with the surrounding cartilage was achieved in adult sheep 5 months postoperatively. In fetal sheep, 28 days after surgery, the defect was filled with differentiating hyaline cartilage in about 80-90% of the repair tissue and 10-20% showed progressing hyalinization and full integration with the surrounding cartilage.

### Injury- and repair-associated macroscopic and histological changes in adult and fetal sheep 3 days postinjury

Upon harvest 3 days postoperatively, the OARSI macroscopic score was 4/16 for cartilage, because of the surgically induced defect in the medial femoral condyle, and 0 for osteophytes (owing to the short time since surgery, no OARSI score was assigned to the macroscopic appearance of the synovium). Within the first 3 days after injury, no histologically visible cartilage repair could be detected. Therefore, none of the established repair scoring systems could be applied. Instead, the description of the structural conditions was based on the evaluation criteria of the International Cartilage Repair Society (ICRS) assessment including the cartilage surface and matrix, cell distribution, cell viability and subchondral bone, but without scores (Mainil-Varlet et al., 2003).

**Fig. 3.**
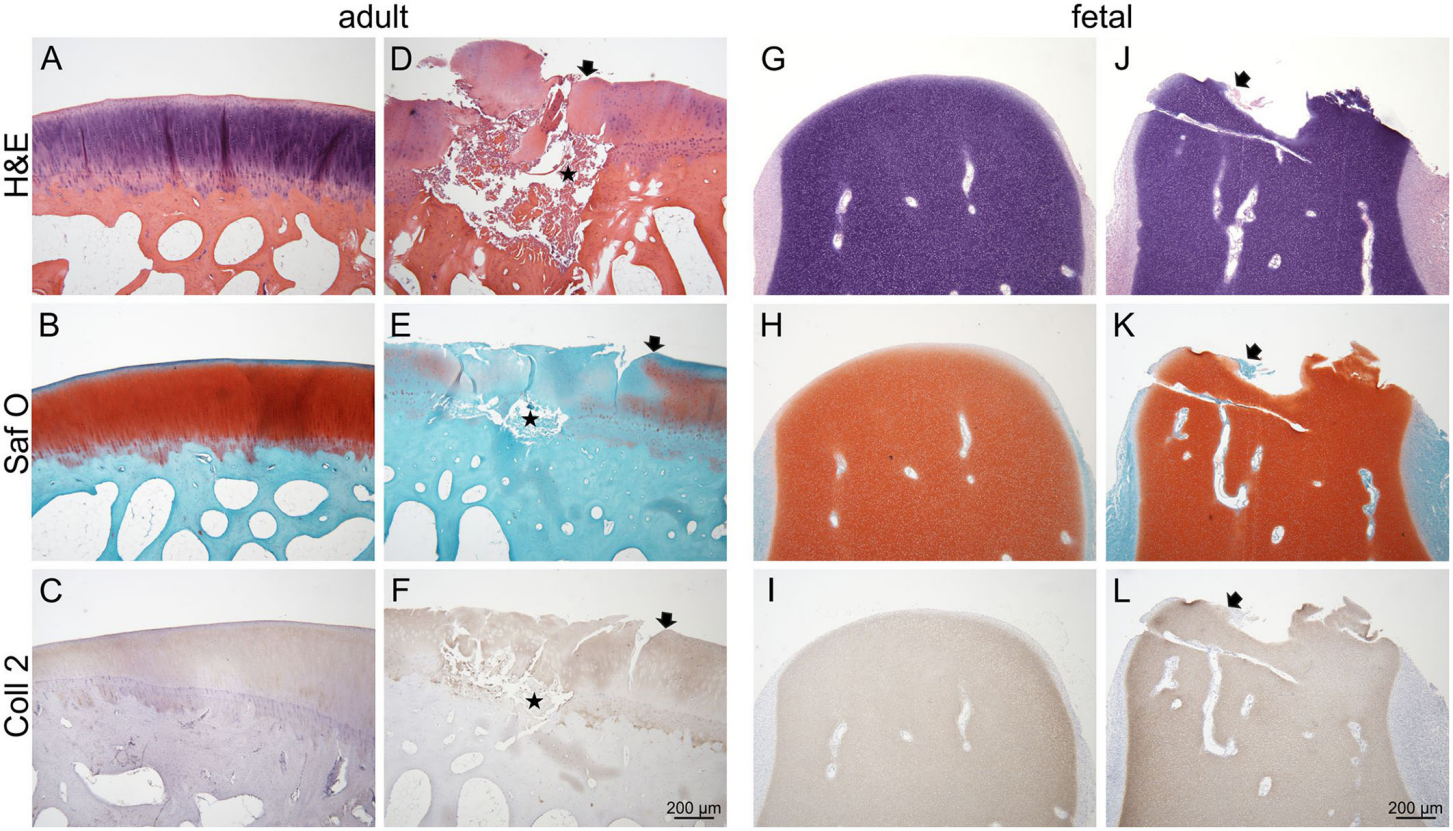
**Morphology and extracellular matrix composition of healthy and injured adult and fetal cartilage: H&E, Safranin O and COL2 staining.** Condyles are shown from the four study groups: (A-C) uninjured adult controls; (D-F) injured adult cartilage; (G-I) uninjured fetal controls; and (J-L) injured fetal cartilage. Arrows mark the transition from healthy cartilage to the lesion site; asterisks indicate sites of microfracture penetrating the subchondral bone plate (D-F). Fibrous tissue partly covering the surface of the lesion (arrows, J-L) was found in fetal injured condyles.

Adult control condyles showed healthy articular cartilage with a smooth surface, physiological matrix composition, and typical distribution of chondrocytes (Fig. 3A,B). COL2 staining was homogeneous and distinct throughout the whole articular cartilage (Fig. 3C).

Creation of the cartilage lesion in adults resulted in matrix depletion at the site of injury as well as in the superficial zone (Fig. 3D,E). Next to the cartilage lesion an acellular area of about 100 μm thickness was found with either empty lacunae or homogenous matrix lacking apparent lacunae. No cell clustering was observed. The microfractures penetrating the subchondral bone plate were visible (Fig. 3D,E). One sample showed a focal accumulation of granulocytes in the bone marrow cavity below the cartilage lesion. In the immediate vicinity (∼10 μm) of the cartilage lesion, COL2 staining intensity was decreased (Fig. 3F).

Similar to the adults, fetal uninjured control samples showed a smooth cartilage surface, homogenous matrix composition, and distinct COL2 staining throughout the whole condyles (Fig. 3G-I).

Although matrix depletion was also detected around the cartilage lesions in the fetal samples, it was less marked compared with adults (Fig. 3J,K). An almost acellular area of 50 μm surrounded the cartilage lesion. The lesion surface was partly covered with fibrous tissue originating either from cartilage canals or connective tissue flanking the articular surface. The pattern of the COL2 staining around the fetal cartilage lesions was similar to that of adults with a 10 μm zone of decreased staining intensity (Fig. 3L).

**Fig. 4.**
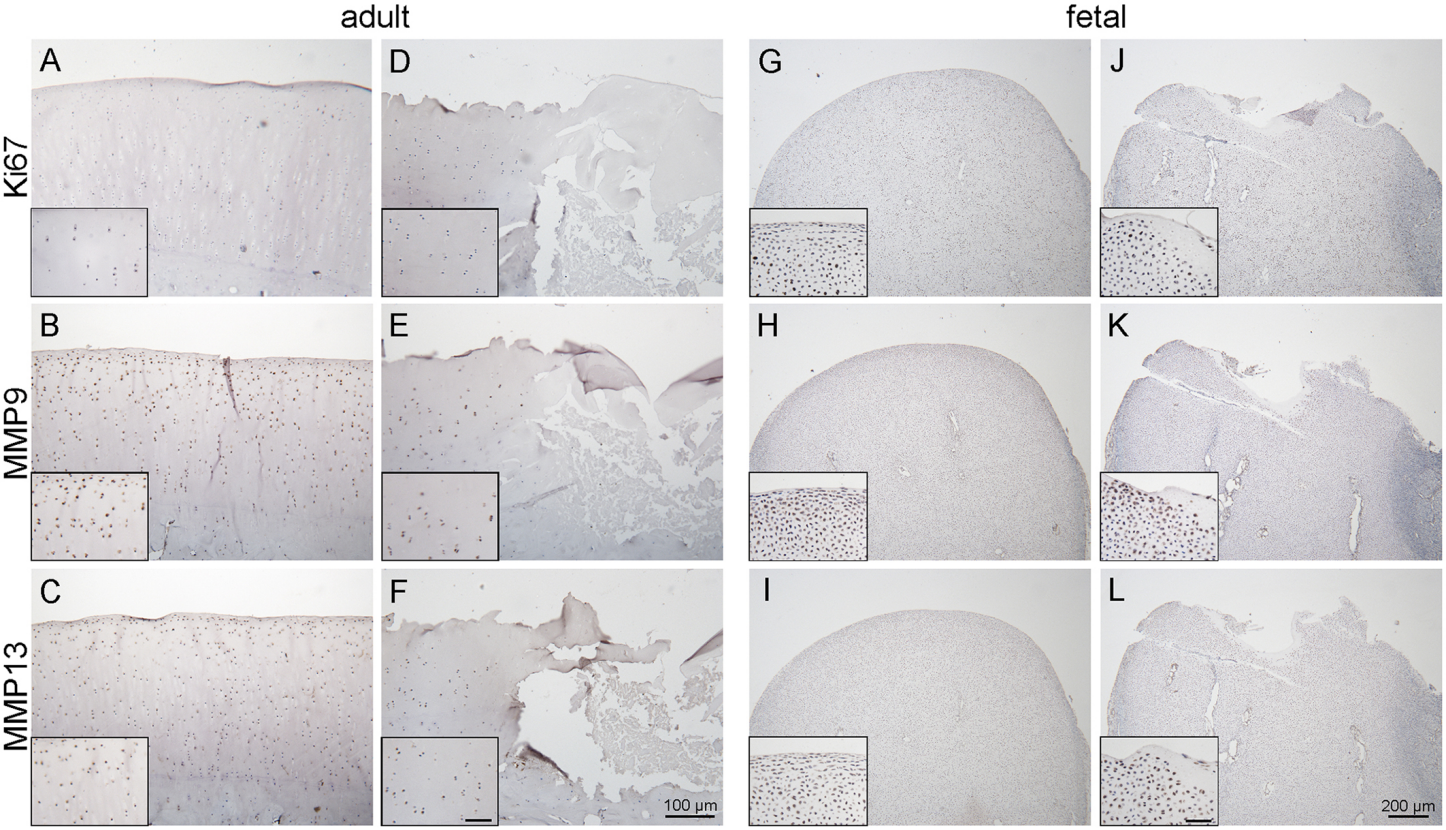
**Distribution of proliferation marker Ki67 and MMPs in healthy and injured adult and fetal cartilage.** (A-C) Uninjured adult controls, (D-F) injured adult cartilage, (G-I) uninjured fetal controls and (J-L) injured fetal cartilage. Inserts in fetal injured condyles indicate transition from healthy cartilage to the lesion site. Scale bars: 100 μm (adult samples); 200 μm (fetal samples); 20 μm (inserts).

In adult control animals, no proliferating cells (demonstrated by expression of the proliferation antigen Ki67) were found in the articular cartilage or the subchondral bone (Fig. 4A). Few Ki67-positive cells were detected in the bone marrow cavities. Chondrocytes in all cartilage zones expressed the matrix metalloproteinases MMP9 and MMP13 with a stronger staining intensity for MMP9 (Fig. 4B,C); however, no MMP-positive osteocytes were observed.

Likewise, in the injured adult cartilage samples, no Ki67-positive cells were observed (Fig. 4D); however, in one sample, an accumulation of Ki67-positive cells was found in a microfracture gap, which was filled with bone marrow. Both MMP9 and MMP13 expression were reduced within and adjacent to the cartilage lesions (Fig. 4E,F), as compared with the intact cartilage of the respective sample.

In fetal healthy cartilage, evenly distributed Ki67-positive cells (Fig. 4G) and MMP-expressing cells (Fig. 4H,I) were detected throughout the whole cartilage. The staining pattern of MMP9 appeared identical to that of MMP13.

Although in the fetal injured cartilage an almost cell-free zone of 50 μm was found to surround the lesions, single Ki67-expressing cells as well as MMP-positive cells could still be detected in this area (Fig. 4J-L). More MMP-expressing cells were located adjacent to the cell-free zone, as well as in the cartilage canals of the injured condyle.

### The ovine model supports comprehensive molecular profiling by high-resolution mass spectrometry

**Fig. 5.**
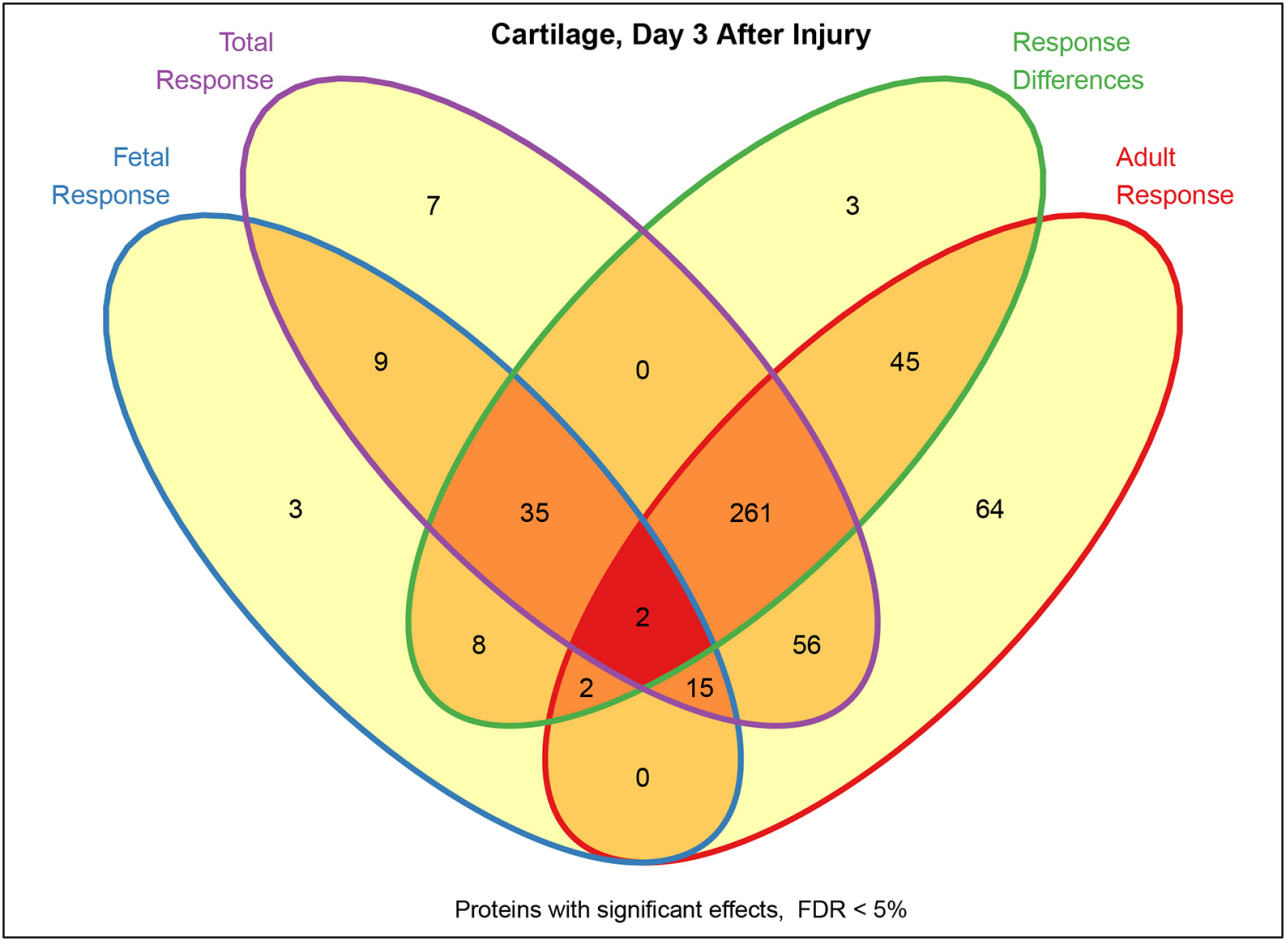
**Venn diagram giving an overview of genes implicated by a range of differential screening tests (*n*=3 biological replicates/group, two technical replicates/biological replicate).** Specifically, we examine the average total response to injury (magenta), the fetal response (blue), the adult response (red), and significant response differences (green). Separately assessing significances for each of the four tests improves sensitivity and specificity by avoiding an accumulation of thresholding artifacts. Comparing cartilage on day 3 after injury with matching control tissues yielded 385 genes implicated in the total response incorporating the average evidence from all sample types (7+9+0+35+261+2+56+15). Analogously, 74 genes were implicated in the fetal response (9+3+35+8+2+2+15), of which 13 were newly identified (3+8+2+0). Conversely, 445 genes were implicated in the adult response (45+261+64+2+56+2+15), of which 111 were newly identified (45+64+2+0). Response differences are shown by 356 genes with an injury response in fetal samples that was significantly different to that in adult samples (3+0+45+35+261+2+8+2), including three genes previously not implicated, eight genes so far only implicated in the fetal response, 45 genes so far only implicated in the adult response, two genes already implicated in both, 35 genes already implicated in the total and the fetal responses, 261 genes already implicated in the total and the adult responses, and two genes implicated in all. These observations reflect the finding that response strength and direction of expression change can be affected differently in the injury response of fetal and adult samples.

Secretome analysis of control and injured (3 days postoperative) cartilage tissue samples derived from adult and fetal sheep, using high-resolution mass spectrometry (MS), enabled the identification of a total number of 2106 distinct proteins. Of these, 445 proteins were found to be significantly regulated (*q*-value <0.05) in response to cartilage injury in adult animals, compared with 74 proteins in fetal animals (Fig. 5). Comparing protein baseline expression, 1288 proteins were found to be significantly differentially regulated between fetal and adult control animals. The injury response of fetal and adult sheep was significantly differentially regulated in 356 proteins. A comparison of protein regulation in adult and fetal animals (Fig. 5, Table 1, Tables S1,S2) revealed differences in three key groups of proteins: (i) proteins associated with the immune response and inflammation, (ii) proteins specific for cartilage tissue and cartilage development, and (iii) proteins involved in cell growth and proliferation (Table 1, Tables S1,S2). Multiple well-known factors in inflammatory processes, such as S100A8, S100A9, S100A12 and CCDC88A, were found to be significantly upregulated following injury in adult (*q*<0.001) but not in fetal animals (Table 1). By contrast, several proteins with anti-inflammatory and growth-supporting effects, such as protein phosphatase Mg^2+^/Mn^2+^-dependent 1A (PPM1A) and cell-division cycle 42 (CDC42), showed a significant increase in response to injury in fetal sheep (*q*=0.005 and 0.006) compared with adults (Table 1). Cartilage-specific proteins, such as aggrecan (ACAN), cartilage oligomeric matrix protein (COMP), chondroadherin (CHAD) and proteoglycan 4 (PRG4), had a significantly higher baseline expression in adults (*q*<0.001) and showed little injury response in either age group with the exception of PRG4, which was significantly upregulated in fetal injured sheep (*q*=0.01). Other proteins related to cell growth and proliferation, such as mitogen-activated protein kinase 3 (MAPK3/ERK1) and GA-binding protein transcription factor α subunit (GABPA), also displayed differences in abundance (*q*=0.02 and 0.04) as well as regulation between adult and fetal sheep (*q*=0.003 and 0.0001).

**Table 1.**
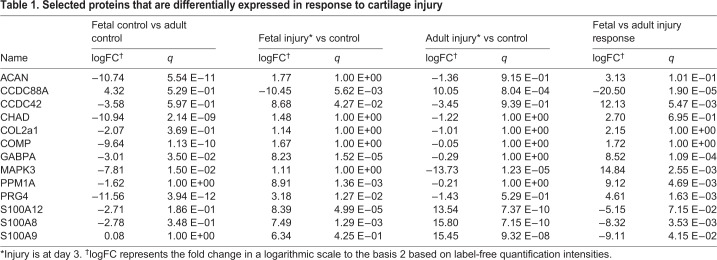
**Selected proteins that are differentially expressed in response to cartilage injury**

**Fig. 6.**
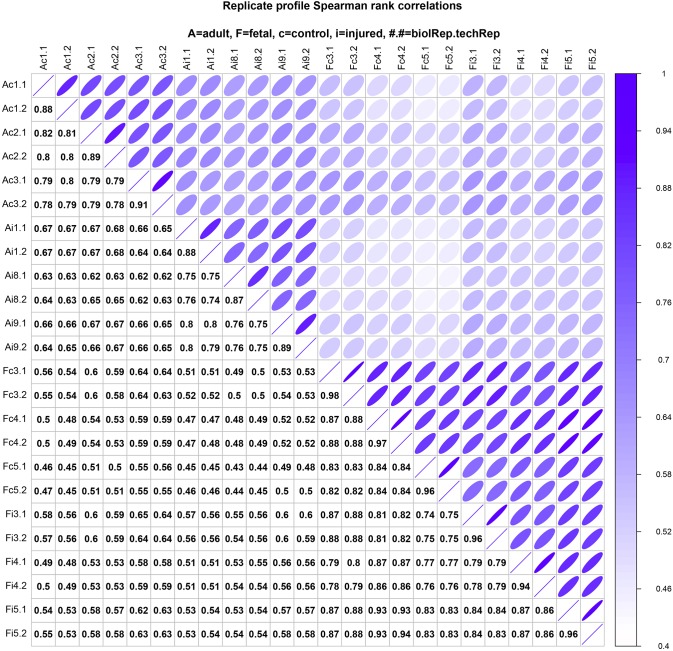
**Sample correlation structure.** This figure compares pairwise sample correlations: Spearman rank correlation coefficients are given in the boxes below the diagonal and correlations are visualized above the diagonal (darker and narrower ellipses indicate higher correlations). Rows and columns show sample labels, where A/F=adult/fetal, c/i=control/injured and #.# show biological and technical replicate numbers (*n*=3 biological replicates/group, two technical replicates/biological replicate).

Our results demonstrate the biological relevance and reproducibility of our new ovine cartilage defect model and MS analysis (Fig. 6). Technical measurement reproducibility was excellent, with variation clearly lower than variation between biological replicates, indicating a high sensitivity of the proteomics profiling workflow (Fig. 6). The robustness of our new cartilage defect model is reflected in the variance across biological replicates being small in relation to the examined biological effects, whether injury versus control or differences between adult and fetal samples (Fig. 6). For both adult and fetal samples, low variance across replicates indicates good reproducibility of our experimental setup, confirming that biologically meaningful signals can sensitively be obtained from only a moderate sample size. Furthermore, our findings confirm good standardization of articular cartilage defects between individuals of both the adult and fetal age group.

## DISCUSSION

Fetal scarless regeneration is a paradigm for ideal tissue repair. The mechanisms of this tightly regulated process, involving the interplay of growth factors, cytokines, proteinases and cellular mediators combined with differences in cellular density, proliferation rate, inflammatory response, extracellular matrix (ECM) composition and synthetic function, are poorly understood compared with adults (Cowin et al., 1998; Degen and Gourdie, 2012; Ferguson and O'Kane, 2004). Studies in dermal wound healing identified qualitative, quantitative and temporal differences in growth factor and cytokine expression between adult and fetal wounds (Cowin et al., 1998; Degen and Gourdie, 2012; Ferguson and O'Kane, 2004).

Our results illustrate the biological relevance, the technical feasibility and repeatability of a new ovine cartilage defect model (Fig. 1) and analytical approaches and confirm regeneration in fetal sheep versus scarring repair in adult sheep (Fig. 2). The well-characterized ontogeny of the ovine immune and inflammatory system and bone marrow niche made the sheep an ideal model in which to examine fetal regeneration (Almeida-Porada et al., 2004; Jeanblanc et al., 2014). The sheep is also a well-accepted and validated model for musculoskeletal disorders and, in particular, cartilage degeneration (Dorotka et al., 2005; Entrican et al., 2015; Mrugala et al., 2008; van Turnhout et al., 2010a,b). Hence, sheep are commonly used to study therapies for OA and articular cartilage lesions (Lu et al., 2006; Munirah et al., 2007; Xue et al., 2013). In addition, fetal sheep share many important physiological and developmental characteristics with humans and have proven themselves invaluable models for mammalian physiology (Almeida-Porada et al., 2004; Jeanblanc et al., 2014). Results obtained in the fetal lamb have been directly applicable to the understanding of human fetal growth and development and are highly predictive of clinical outcome in a variety of applications including *in utero* stem-cell transplantation (Almeida-Porada et al., 2004, 2007; Jeanblanc et al., 2014; Kim et al., 2013; Kuypers et al., 2012; Liechty et al., 2000; Porada et al., 2005). A number of specific characteristics make sheep particularly well-suited for OA, regenerative medicine and fetal regeneration research enabling results of high clinical relevance to be obtained: (1) large size facilitating repeated sampling from individual animals and harvest of adequate sample sizes; (2) comparable body weight to humans; (3) similar mechanical exertion to humans (Bruns et al., 2000; Russo et al., 2015); (4) relatively long life expectancy (lifespan 8-12 years) allowing longitudinal analysis as well as evaluation of long-term efficacy and safety of treatments; (5) long gestational period (150 days) provides sufficient temporal resolution to translate findings obtained in sheep into human parameters (Jeanblanc et al., 2014); (6) extremely well-characterized immune development analogous to humans (Almeida-Porada et al., 2004; Jeanblanc et al., 2014; Maddox et al., 1987; Miyasaka and Trnka, 1986; Osburn, 1981; Sawyer et al., 1978); (7) bone marrow ontogeny and niche development closely paralleling humans (Jeanblanc et al., 2014).

Furthermore, for the sheep model, an acceptable annotation status and representative subsets of identified proteins are available on sources such as the National Center for Biotechnology Information (NCBI; e.g. 30,584 genes and 69,889 proteins) (NCBI Resource Coordinators, 2018), allowing good applicability and translation of the results.

In this study, we compared the adult and fetal response to cartilage injury 3 days after lesion induction as this time point is established to be within the time window of inflammation for both adult and fetal individuals, one of the injury responses hypothesized to crucially contribute to the difference between adult and fetal healing. For the fetal injury response, it is only known that cartilage regeneration occurs within 4 weeks. This is in stark contrast to the adult injury response, which comprises an inflammatory phase of 3-5 days, a proliferative phase of 3-6 weeks and a remodeling phase of up to 1 year duration resulting in a fibrocartilaginous scar. As the timeline of the fetal injury response is not yet established, choosing a later date would have made data interpretation and correlation of adult and fetal data impossible. Three days is within the peak period of the adult inflammatory response, allows for recruitment of monocytes/macrophages to the injury site and has also been shown to be associated with signs of inflammation in fetal injuries in other tissues.

The main factors identified within the secretome were ECM proteins, growth factors and inflammatory mediators such as cytokines and chemokines. Considering the key chondrocyte signaling pathways regulating processes of inflammation, cell proliferation, differentiation and matrix remodeling, which include the p38, JNK and ERK mitogen-activated protein (MAP) kinases, the phosphatidylinositol 3-kinase (PI3K)-AKT pathway, the JAK-STAT pathway, Rho GTPases and WNT–β-catenin and SMAD pathways (Beier and Loeser, 2010), our data provide an initial indication of differences in the inflammatory response to injury between adult and fetal cartilage and suggest the active production of anti-inflammatory and growth factors, such as PPM1A and CDC42, in the fetal environment.

PPM1A is a bona fide phosphatase, which is involved in the regulation of many developmental processes such as skeletal and cardiovascular development. Through its role as phosphatase of many signaling molecules, such as p38 kinase, CDK2, PI3K, Axin and SMAD, upregulation of PPM1A abolishes, for example, transforming growth factor β (TGF-β)-induced antiproliferative and transcriptional responses (Wang et al., 2014), as well as bone morphogenetic protein (BMP) signaling (Duan et al., 2006). Furthermore, through the dephosphorylation of IκB kinase β, PPM1A terminates tumor necrosis factor α (TNFα)-induced NF-κB activation and is thus involved in the regulation of inflammation, the immune response and apoptosis (Sun et al., 2009).

CDC42 belongs to the family of Rho GTPases and controls a broad variety of signal transduction pathways regulating cell migration, polarization, adhesion proliferation, differentiation and apoptosis in a variety of cell types (Sun et al., 2009). CDC42 is required in successive steps of chondrogenesis by promoting mesenchymal condensation through the BMP2/CDC42/PAK/p38/SMAD signaling cascade and chondrogenic differentiation through the TGF-β/CDC42/PAK/AKT/SOX9 signaling pathway (Wang et al., 2016). Another essential CDC42 function relevant to the current study is its involvement in wound healing by attenuating MMP1 expression (Rohani et al., 2014) and regulating spatially distinct aspects of the cytoskeleton machinery, especially actin mobilization toward the wound (Benink and Bement, 2005). Given the increase of actin-containing articular chondrocytes in response to cartilage injury, these could also have a role in the healing of cartilage defects (Wang et al., 2000).

By contrast to the anti-inflammatory factors upregulated in fetal sheep in response to injury, adult sheep displayed a significant increase of inflammatory mediators such as alarmins S100A8, S100A9, S100A12 and CCDC88A. The alarmin S100 proteins are markers of destructive processes such as those occurring in OA (Liu-Bryan and Terkeltaub, 2015; Nefla et al., 2016; van den Bosch et al., 2015). Accordingly, in OA articular S100A8 and S100A9 protein secretion is increased, recruiting immune cells to inflamed synovia, initiating the adaptive immune response, inducing canonical WNT signaling and promoting cartilage matrix catabolism, osteophyte formation, angiogenesis and hypertrophic differentiation (Liu-Bryan and Terkeltaub, 2015; Nefla et al., 2016; van den Bosch et al., 2015). S100A8 and S100A9 upregulate markers characteristic for ECM degradation [MMPs 1, 3, 9 and 13, interleukin 6 (IL-6) and IL-8] and downregulate growth promotion markers (ACAN and COL2); thus, these alarmins have a destructive effect on chondrocytes, causing proteoglycan depletion and cartilage breakdown (Schelbergen et al., 2012). Also, S100A12 is upregulated in OA cartilage and has been shown to increase the production of MMP13 and vascular endothelial growth factor (VEGF) in OA chondrocytes via the p38 MAPK and NF-κB pathways (Nakashima et al., 2012). Another relevant protein, which was significantly downregulated upon injury in fetal sheep but significantly upregulated in injured adult sheep, is CCDC88A. CCDC88A is a multimodular signal transducer, which modulates growth factor signaling during diverse biological and disease processes including cell migration, chemotaxis, development, self-renewal, apoptosis and autophagy. CCDC88A exerts its effect on these processes by integrating signals downstream of a variety of growth factors, such as epidermal growth factor, insulin-like growth factor, VEGF, insulin, STAT3, platelet-derived growth factor receptor and trimeric G-protein Gi (Dunkel et al., 2012; Ghosh et al., 2008). In addition, CCDC88A, which is expressed at high levels in immune cells of the lymphoid lineage, has an important role in T-cell maturation, activation and cytokine production during pathological inflammation and its inhibition could help treat inflammatory conditions, as shown *in vitro* and in mouse studies (Kennedy et al., 2014). Furthermore, CCDC88A, through activation of Gαi, simultaneously enhances the profibrotic (PI3K-AKT-FOXO1 and TGF-β-SMAD) and inhibits the antifibrotic (cAMP-PKA-pCREB) pathways, shifting the fibrogenic signaling network towards a profibrotic programme (Lopez-Sanchez et al., 2014). Interestingly, in the liver, sustained upregulation of CCDC88A occurs in all forms of chronic fibrogenic injuries, but not in acute injuries that heal without fibrosis. This observation indicates that increased expression of CCDC88A during acute injuries might enhance progression to chronicity and fibrosis (Lopez-Sanchez et al., 2014). CCDC88A also regulates the PI3K-AKT pathway, which exhibits pleiotropic functions in chondrogenesis, cartilage homeostasis and inflammation. This pathway might further induce an increase in MMP production by chondrocytes leading to subsequent cartilage degeneration, through its multiple downstream target proteins (Chen et al., 2013; Fujita et al., 2004; Greene and Loeser, 2015; Kita et al., 2008; Litherland et al., 2008; Starkman et al., 2005; Xu et al., 2015).

Remarkably, in this study, the cartilage matrix proteins PRG4, ACAN, COMP and CHAD had a significantly higher baseline expression in adult sheep and showed little injury response in either age group with the exception of PRG4, which was significantly upregulated in fetal injured sheep. PRG4, in response to injury, increased 3.2-fold (*q*=0.01) in fetal sheep, which is a 4.6-fold higher increase compared with adults (*q*=0.002), indicating a stronger and more rapid cartilage matrix production. As PRG4-expressing cells constitute a cartilage progenitor cell population, the higher baseline expression in adults is particularly surprising. This observation can be explained, however, by the restriction of PRG4 to the most superficial cell layer in fetal joints compared with a distribution throughout the entire cartilage depth in older individuals (Kozhemyakina et al., 2015).

By contrast to the cartilage matrix glycoproteins, many growth factors such as GABPA and MAPK3 showed, as expected, differential regulation following injury between adult and fetal sheep. GABPA, a member of the ETS protein family which is ubiquitously expressed and has an essential role in cellular functions such as cell-cycle regulation, cellular growth, apoptosis and differentiation (Rosmarin, 2004), showed a further significant upregulation in fetal injury and no response to adult injury (*q*=0.0001). GABPA activates the transcriptional co-activator Yes-associated protein (YAP), which is essential for cellular and tissue defences against oxidative stress, cell survival and proliferation and can induce the expression of growth-promoting genes important for tissue regeneration after injury (Wen Chun Juan, 2016). The cellular importance of GABPA is further highlighted by the observation that in *Gabpa* conditional knockout embryonic stem cells (ESCs), disruption of GABPA drastically repressed ESC proliferation and cells started to die within 2 days (Ueda et al., 2017).

The growth regulator MAPK3 had a higher baseline expression in adult sheep (logFC=7.8, *q*=0>02), but significantly decreased (13.7 logFC, *q*<0.0001) after injury; fetal MAPK3 remained essentially unchanged. MAPK3 is an essential component of the MAP kinase signal transduction pathway and as such contributes to cell growth, adhesion, survival and differentiation through the regulation of transcription, translation and cytoskeletal rearrangements. MAPK3 also fulfils an essential role in the control of chondrogenesis and osteogenesis of mesenchymal stem cells (MSCs) under TGF-β or mechanical induction and positively regulates chondrogenesis of MSCs (Arita et al., 2011; Pelaez et al., 2012; Bobick et al., 2010).

Our results are consistent with previous studies in fetal skin wounds, which have also shown a different and reduced inflammatory response and decreased scar formation during dermal wound healing in fetal sheep and mice (Cowin et al., 1998). The chronic progressive articular damage of OA is associated with similar levels of pro-inflammatory cytokines and chemokines throughout the disease (Ritter et al., 2013) and occurs through a complex programme involving on-going local inflammation triggered by cytokines and endogenous activation of innate immunity, complement and metabolic pathways (Pap and Korb-Pap, 2015). Therefore, the different inflammatory response demonstrated here in the fetus might be a major contributor to fetal scarless cartilage healing. This is especially intriguing as fetal sheep have a normally functioning immune system by 75 gd (Almeida-Porada et al., 2004; Emmert et al., 2013). Leukocytes have been shown to be present and increase rapidly at the end of the first trimester (Mackay et al., 1986; Maddox et al., 1987). Fetal sheep are able to form large amounts of specific antibodies in response to antigen stimuli by 70 gd (Silverstein et al., 1963) and reject orthotopic skin grafts and stem cell xenotransplants administered after 75-77 gd with the same competence and rapidity as adults (Silverstein et al., 1964). Furthermore, fetal sheep have an inflammatory response to injury before 80 gd (Kumta et al., 1994; Moss et al., 2008; Nitsos et al., 2006). The first evidence of inflammation, the presence of TNF and IL-1, has even been shown as early as 30-40 gd (Dziegielewska et al., 2000).

In conclusion, our results demonstrate the power of a new ovine fetal cartilage regeneration model and of our analytical approach. A comparison of fetal and adult protein regulation in response to cartilage injury found both positive and negative regulators of inflammatory events to be differentially regulated. These findings hold promise for potential therapeutic interventions, as the presence of a negative regulator is more easily mimicked than the absence of a positive regulator. Further studies employing this newly developed animal model and using analytical techniques to identify proteins involved in OA aetiology and pathogenesis, as well as potential biomarkers and therapeutic targets, are warranted. Such studies will enable us to work towards the goal of finding novel biomimetic solutions, which might be exploited to favourably shift the adult cartilage healing milieu to a more fetal phenotype to induce regeneration by recapitulating cartilage ontogeny.

## MATERIALS AND METHODS

### Animal model

Standardized cartilage lesions were created in musculoskeletally mature (2-5 years, body weight 95±12 kg), female, healthy, non-gravid Merino-cross ewes (*Ovis* *aries*) without orthopaedic disease and fetal lambs (80 gd, term=∼150 days) with approval of the national (Federal Ministry of Science, BMWFW) and institutional animal welfare committee (approval numbers 68.205/0155-WF-/V/3b/2014 and 68.205/0028-II/3b/2014). For the fetal subjects, only twin pregnancies were included to provide a twin lamb as uninjured control on a background of low genetic variation to allow differentiation between protein secretion of regular fetal development and fetal response to cartilage injury. Fetal hind limbs were exteriorized from the uterus via a standard ventral-midline laparotomy followed by an uterotomy over a randomly chosen uterine horn.

For the purpose of lesion induction, a minimally invasive medial parapatellar arthrotomy (Orth and Madry, 2013) was performed in both knees in adult and fetal sheep. A bilateral full-thickness cartilage lesion with a diameter of 7 mm (adult sheep) and 1 mm (fetal lamb) was created in the medial femoral condyle region (Fig. 1) using a custom-made precision surgical instrument (trocar with sleeve) for adult sheep and for fetal lambs a Versi-handle (Ellis Instruments, Madison, NJ, USA) with adjustable depth control, which was set at a lesion depth of 1 mm.

To ensure that differences in the healing response between fetal and adult articular cartilage are not caused by differences in blood supply, which in fetal sheep is starting at an articular cartilage depth of 400 μm (Namba et al., 1998), we created three microfractures through the subchondral bone plate in adult cartilage defects to gain access to the bone marrow vasculature (Dorotka et al., 2005). The arthrotomy was closed in one dermal layer using 6-0 monofilament nylon (Monosof, Covidien, Minneapolis, MN, USA) in fetal lambs and in three layers using 2-0 monofilament absorbable polyester (Biosyn, Covidien) for the joint capsule and subcutaneous tissue and 2-0 monofilament nylon for the skin in adult sheep. Adult animals were allowed full weight-bearing immediately after surgery. All adult sheep were treated with antibiotics peri-operatively and received pain management. Pain management was provided with morphine to avoid anti-inflammatory drugs, which would influence the result of the study.

### Pilot study

In a pilot study, as a proof-of-principle of fetal regeneration versus adult fibrocartilaginous repair, three adult and three fetal injured sheep were euthanized at 5 months (adult) and 28 days (fetal) postoperatively for macroscopic and histological evaluation of the defect repair. At the time of euthanasia, digital photographs were taken and the articular cartilage surface and the cartilage defect healing response was macroscopically evaluated using the OARSI recommendations for macroscopic scoring of cartilage damage in sheep, taking into account surface roughening, fibrillation, fissures and the presence and size of osteophytes and erosions (Little et al., 2010). For fetal sheep the OARSI macroscopic score was size-adjusted by multiplying the adult lesion size cut-off values with 3.4/36.4, the ratio of the reported tibia length of fetal versus adult sheep (Mufti et al., 2000; Salami et al., 2011).

### Tissue harvest

At day 3 after injury, samples were collected from three biological replicates per comparison group (adult injured, fetal injured, fetal uninjured twin control). In adult sheep, samples were also harvested from uninjured controls (*n*=3).

After macroscopic scoring, the medial femoral condyles were surgically excised and left and right knees were randomly assigned to MS and histology. For MS the (cartilage) tissue remnants contained in the defect area and the cartilage rim surrounding the lesion (3 mm width in adults; 1 mm width in fetal sheep) were excised.

### Histology and immunohistochemistry

For histological analysis the femoral condyles were fixed in 4% buffered formalin. Condyles from adult sheep were decalcified in 8% neutral EDTA. After embedding in paraffin, 4 µm thick sections were cut and mounted on 3-aminopropyltriethoxysilane (APES)-glutaraldehyde-coated slides (Sigma-Aldrich, Vienna, Austria). Consecutive sections were stained with Haematoxylin and Eosin (H&E) and Safranin O (Mulisch and Welsch, 2015).

For immunohistochemistry, sections were deparaffinized, rehydrated and endogenous peroxidase was blocked with 0.6% hydrogen peroxide in methanol (15 min at room temperature). Nonspecific binding of antibodies was prevented by incubation with 1.5% normal goat serum (Dako Cytomation, Glostrup, Denmark) in phosphate-buffered saline (PBS; 30 min at room temperature). Primary antibodies (anti-COL2, anti-Ki67, anti-MMP9 and anti-MMP13; Table 2) were incubated overnight at 4°C. An appropriate BrightVision Peroxidase system (Immunologic, Duiven, The Netherlands) was used and peroxidase activities were localized with diaminobenzidine (DAB; Sigma-Aldrich). Cell nuclei were counterstained with Mayer's Haematoxylin.

**Table 2.**
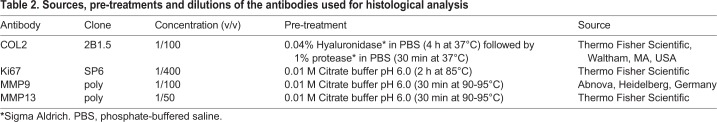
**Sources, pre-treatments and dilutions of the antibodies used for histological analysis**

Tissue from adult sheep mammary glands and human placenta served as positive controls. For negative controls, the primary antibody was omitted.

### Mass spectrometry

The cartilage rim and the tissue remnants obtained from the lesion site were cultivated in serum-free RPMI medium (Gibco, Life Technologies, Austria) supplemented with 100 U/ml penicillin and 100 µg/ml streptomycin (ATCC, LGC Standards GmbH, Germany) for 6 h under standard cell-culture conditions (37°C and 5% CO_2_). Afterwards, medium was sterile-filtered through a 0.2 µm filter and precipitated overnight with ice-cold ethanol at −20°C. After precipitation, proteins were dissolved in sample buffer [7.5 M urea, 1.5 M thiourea, 4% CHAPS, 0.05% SDS, 100 mM dithiothreitol (DDT)] and protein concentrations were determined using Bradford assay (Bio-Rad Laboratories, Munich, Germany).

A 20 μg sample of each protein was used for a filter-aided digestion, as described previously (Aukland, 1991; Aukland et al., 1997a,b; Bileck et al., 2014; Wiśniewski et al., 2009). Briefly, 3 kDa molecular weight cut-off filters (Pall Austria Filter GmbH) were conditioned with MS-grade water (Millipore GmbH). Protein samples were concentrated on the pre-washed filter by centrifugation at 15,000 ***g*** for 15 min. After reduction with DTT [5 mg/ml dissolved in 8 M guanidinium hydrochloride in 50 mM ammonium bicarbonate (ABC) buffer, pH 8] and alkylation with iodoacetamide (10 mg/ml in 8 M guanidinium hydrochloride in 50 mM ABC buffer), samples were washed and 1 µg trypsin was added before incubation at 37°C for 18 h. After enzymatic digestion, peptide samples were cleaned with C18 spin columns (Pierce, Thermo Scientific, Germany), dried and stored at −20°C until analysis.

For mass spectrometric analyses, dried samples were reconstituted in 5 µl 30% formic acid (FA) containing 10 fmol each of four synthetic standard peptides and diluted with 40 µl mobile phase A (H_2_O:ACN:FA=98:2:0.1). A 10 µl aliquot of the peptide solution was loaded onto a 2 cm×75 µm C18 Pepmap100 precolumn (Thermo Fisher Scientific, Austria) at a flow rate of 10 µl/min using mobile phase A. Afterwards, peptides were eluted from the precolumn to a 50 cm×75 µm Pepmap100 analytical column (Thermo Fisher Scientific, Austria) at a flow rate of 300 nl/min and separation was achieved using a gradient of 8% to 40% mobile phase B (ACN:H_2_O:FA=80:20:0.1) over 95 min. For mass spectrometric analyses, MS scans were performed in the range of *m/z* 400-1400 at a resolution of 70,000 (at *m/z*=200). MS/MS scans of the eight most abundant ions were achieved through high-energy collisional dissociation fragmentation at 30% normalized collision energy and analysed in the orbitrap at a resolution of 17,500 (at *m/z*=200). All samples were analysed in duplicate.

### Data analysis and statistics of MS experiments

Protein identification and label-free quantitative (LFQ) data analysis were performed using the open source software MaxQuant 1.3.0.5 including the Andromeda search engine (Cox and Mann, 2008). Protein identification was achieved searching against *Ovis aries* in the Uniprot Database (version 09/2014 with 26,864 entries) allowing a mass tolerance of 5 ppm for MS spectra and 20 ppm for MS/MS spectra, as well as a maximum of two missed cleavages. In addition, carbamidomethylation on cysteine residues was included as a fixed modification, whereas methionine oxidation and N-terminal protein acetylation were included as variable modifications. Furthermore, search criteria included a minimum of two peptide identifications per protein, at least one of them unique, and the false discovery rate (FDR) calculation based on *q*-values, performed for both peptide identification as well as protein identification, less than 0.01. Prior to statistical analyses, proteins were filtered for reversed sequences, contaminants and a minimum of three independent identifications per protein.

Missing values were replaced by a global *ε*, set to the minimum intensity observed in the entire data set divided by four. This sensitivity-based pseudo-count reflects the prior belief of non-observed protein expression, maintaining a lower bound of a fourfold change for differences to proteins not observed in one sample group, thus maintaining sensitivity while improving specificity by mitigating the effects of random non-observations. The Spearman rank correlations between samples shown in Fig. 6 are not affected by this transform. For the visualization of the sample correlation structure in Fig. 6, ellipses were plotted as (*x*, *y*)=(cos(*θ*+*d*/2), cos(*θ*-*d*/2)), where *θ* ɛ [0,2π) and cos(*d*)=*ρ*, with *ρ* the Spearman rank correlation coefficient (Murdoch and Chow, 1996).

Protein expression profile analysis was performed in the statistical environment R (www.r-project.org). Differential expression contrasts were computed with Bioconductor libraries (www.bioconductor.org). Data were normalized by a mean log-shift, standardizing mean expression levels per sample. Linear models were fitted separately for each protein, computing second-level contrasts for a direct test of differences between fetal and adult responses to injury. Conservative Benjamini-Yekutieli correction was used to adjust for multiple testing to give strong control of the FDR. We call significant features for *q*-values <0.05. Linear models were adjusted for the nested correlation structure of technical and biological replicates (see Fig. 6). Significance was assessed by regularized *t*-tests. For these, group variances are shrunk by an Empirical Bayes procedure to mitigate the high uncertainty of variance estimates for the available sample sizes (Sun et al., 2009). The employed algorithms are implemented in the package limma (Smyth, 2005), which is available from Bioconductor.

## Supplementary Material

Supplementary information
