# Supplementary material for: Fetal articular cartilage regeneration versus adult fibrocartilaginous repair: secretome proteomics unravels molecular mechanisms in an ovine model
Source: Dis Model Mech. 2018 Jul 6;11(7):dmm033092. doi: 10.1242/dmm.033092 (PMC6078409; doi:10.1242/dmm.033092)
Supplement: Supplementary information [file dmm-11-033092-s1.pdf]

Supplementary table 1

Name	Gene ontology annotation functions and processes
ACAN	GO:0031012 extracellular matrix, GO:0005201 extracellular matrix structural constituent, GO:0005515 protein binding, GO:0005540 hyaluronic acid binding, GO:0030246 carbohydrate binding, GO:0046872 metal ion binding, GO:0001501 skeletal system development, GO:0006508 proteolysis, GO:0007155 cell adhesion, GO:0007417 central nervous system development, GO:0018146 keratan sulfate biosynthetic process, GO:0022617 extracellular matrix disassembly, GO:0030198 extracellular matrix organization, GO:0042340 keratan sulfate catabolic process
CCDC88A	GO:0003779 actin binding, GO:0008017 microtubule binding, GO:0035091 phosphatidylinositol binding, GO:0042803 protein homodimerization activity, GO:0043422 protein kinase B binding, GO:0051959 dynein light intermediate chain bindin, GO:0001932 regulation of protein phosphorylationg, GO:0006260 DNA replication, GO:0006275 regulation of DNA replication, GO:0007399 nervous system development, GO:0010975 regulation of neuron projection development, GO:0016477 cell migration, GO:0030030 cell projection organization, GO:0030032 lamellipodium assembly, GO:0030705 cytoskeleton-dependent intracellular transport, GO:0031122 cytoplasmic microtubule organization, GO:0031929 TOR signaling, GO:0032148 activation of protein kinase B activity, GO:0032956 regulation of actin cytoskeleton organization, GO:0042127 regulation of cell proliferation, GO:0045724 positive regulation of cilium assembly, GO:0061024 membrane organization, GO:1903566 positive regulation of protein localization to cilium
CDC42	GO:0005095 GTPase inhibitor activity, GO:0005198 structural molecule activity, GO:0006909 phagocytosis, GO:0007165 signal transduction, GO:0008360 regulation of cell shape, GO:0034260 negative regulation of GTPase activity
CHAD	GO:0004860 protein kinase inhibitor activity, GO:0006469 negative regulation of protein kinase activity, GO:0019221 cytokine-mediated signaling pathway, GO:0046426 negative regulation of JAK-STAT cascade, GO:0060348 bone development, GO:1900155 negative regulation of bone trabecula formation
COL2a1	GO:0005201 extracellular matrix structural, GO:0042289 MHC class II protein binding, GO:0042802 identical protein binding, GO:0046872 metal ion binding, GO:0048407 platelet-derived growth factor binding, GO:0001501 skeletal system development, GO:0001502 cartilage condensation, GO:0001503 ossification, GO:0001894 tissue homeostasis, GO:0001958 endochondral ossification, GO:0002062 chondrocyte differentiation, GO:0003007 heart morphogenesis, GO:0006029 proteoglycan metabolic process, GO:0007417 central nervous system development, GO:0007601 visual perception, GO:0007605 sensory perception of sound, GO:0010468 regulation of gene expression, GO:0030198 extracellular matrix organization, GO:0030199 collagen fibril organization, GO:0030574 collagen catabolic process, GO:0030903 notochord development, GO:0035108 limb morphogenesis, GO:0042472 inner ear morphogenesis, GO:0048705 skeletal system morphogenesis, GO:0048839 inner ear development, GO:0050776 regulation of immune response, GO:0051216 cartilage development, GO:0060021 palate development, GO:0060174 limb bud formation, GO:0060272 embryonic skeletal joint morphogenesis, GO:0060348 bone development, GO:0060351 cartilage development involved in endochondral bone morphogenesis, GO:0071599 otic vesicle development, GO:0071773 cellular response to BMP stimulus, GO:0097065 anterior head development, GO:2001240 negative regulation of extrinsic apoptotic signaling pathway in absence of ligand
COMP	GO:0031012 extracellular matrix, GO:0002020 protease binding, GO:0005201 extracellular matrix structural constituent, GO:0005509 calcium ion binding, GO:0005515 protein binding, GO:0005518 collagen binding, GO:0008201 heparin binding, GO:0001501 skeletal system development, GO:0003417 growth plate cartilage development, GO:0006915 apoptotic process, GO:0007155 cell adhesion, GO:0009887 animal organ morphogenesis, GO:0030198 extracellular matrix organization, GO:0043066 negative regulation of apoptotic process, GO:0060173 limb development

Name	Gene ontology annotation functions and processes
GABPA	GO:0000978 RNA polymerase II proximal promoter sequence-specific DNA binding, GO:0001077 transcriptional activator activity, RNA polymerase II proximal promoter sequence-specific DNA binding, GO:0001228 transcriptional activator activity, RNA polymerase II transcription regulatory region sequence-specific DNA binding, GO:0003677 DNA binding, GO:0003682 chromatin binding, GO:0003700 DNA binding transcription factor activity, GO:0003713 transcription coactivator activity, GO:0005515 protein binding ECO:0000353, GO:0033613 activating transcription factor binding, GO:0043565 sequence-specific DNA binding, GO:0044212 transcription regulatory region DNA binding, GO:0046982 protein heterodimerization activity, GO:0000122 negative regulation of transcription by RNA polymerase II, GO:0001701 in utero embryonic development, GO:0006351 transcription, DNA-templated, GO:0006355 regulation of transcription, DNA-templated, GO:0006357 regulation of transcription by RNA polymerase II, GO:0006366 transcription by RNA polymerase II, GO:0007005 mitochondrion organization, GO:0010628 positive regulation of gene expression, GO:0030154 cell differentiation, GO:0045653 negative regulation of megakaryocyte differentiation, GO:0045944 positive regulation of transcription by RNA polymerase II, GO:1903351 cellular response to dopamine
MAPK3	GO:0000166 nucleotide binding, GO:0001784 phosphotyrosine residue binding, GO:0004672 protein kinase activity, GO:0004674 protein serine/threonine kinase activity, GO:0004707 MAP kinase activity, GO:0005515 protein binding, GO:0005524 ATP binding, GO:0016301 kinase activity, GO:0016740 transferase activity, GO:0019902 phosphatase binding, GO:0042802 identical protein binding, GO:0097110 scaffold protein binding, GO:0000165 MAPK cascade, GO:0000187 activation of MAPK activity, GO:0001934 positive regulation of protein phosphorylation, GO:0002741 positive regulation of cytokine secretion involved in immune response, GO:0006351 transcription, DNA-templated, GO:0006361 transcription initiation from RNA polymerase I promoter, GO:0006461 protein-containing complex assembly, GO:0006468 protein phosphorylation, GO:0006915 apoptotic process, GO:0006974 cellular response to DNA damage stimulus, GO:0006975 DNA damage induced protein phosphorylation, GO:0007049 cell cycle, GO:0007411 axon guidance, GO:0008543 fibroblast growth factor receptor signaling pathway, GO:0008543 fibroblast growth factor receptor signaling pathway, GO:0008543 fibroblast growth factor receptor signaling pathway, GO:0009636 response to toxic substance, GO:0009887 animal organ morphogenesis, GO:0010628 positive regulation of gene expression, GO:0010759 positive regulation of macrophage chemotaxis, GO:0014032 neural crest cell development, GO:0014066 regulation of phosphatidylinositol 3-kinase signaling, GO:0016032 viral process, GO:0016310 phosphorylation, GO:0018105 peptidyl-serine phosphorylation, GO:0019233 sensory perception of pain, GO:0019369 arachidonic acid metabolic process, GO:0030168 platelet activation, GO:0030278 regulation of ossification, GO:0030509 BMP signaling pathway, GO:0030641 regulation of cellular pH, GO:0030878 thyroid gland development, GO:0031281 positive regulation of cyclase activity, GO:0031663 lipopolysaccharide-mediated signaling pathway, GO:0032212 positive regulation of telomere maintenance via telomerase, GO:0032496 response to lipopolysaccharide, GO:0032872 regulation of stress-activated MAPK cascade, GO:0033129 positive regulation of histone phosphorylation, GO:0034198 cellular response to amino acid starvation, GO:0034614 cellular response to reactive oxygen species, GO:0035066 positive regulation of histone acetylation, GO:0035556 intracellular signal transduction, GO:0038083 peptidyl-tyrosine autophosphorylation, Fc-epsilon receptor signaling pathway Reactome:R-HSA-2454202 P

Disease Models & Mechanisms • Supplementary information

Name	Gene ontology annotation functions and processes
	UniProtKB P27361 GO:0038096 Fc-gamma receptor signaling pathway involved in phagocytosis, GO:0043330 response to exogenous dsRNA, GO:0045727 positive regulation of translation, GO:0045944 positive regulation of transcription by RNA polymerase II, GO:0051090 regulation of DNA binding transcription factor activity, GO:0051090 regulation of DNA binding transcription factor activity, GO:0051216 cartilage development, GO:0051403 stress-activated MAPK cascade, GO:0051493 regulation of cytoskeleton organization, GO:0051973 positive regulation of telomerase activity, GO:0060020 Bergmann glial cell differentiation, GO:0060324 face development, GO:0060397 JAK-STAT cascade involved in growth hormone signaling pathway, GO:0060425 lung morphogenesis, GO:0060440 trachea formation, GO:0061308 cardiac neural crest cell development involved in heart development, GO:0070371 ERK1 and ERK2 cascade, GO:0070374 positive regulation of ERK1 and ERK2 cascade, GO:0070498 interleukin-1-mediated signaling pathway, GO:0070849 response to epidermal growth factor, GO:0070849 response to epidermal growth factor, GO:0071260 cellular response to mechanical stimulus, GO:0071276 cellular response to cadmium ion, GO:0071356 cellular response to tumor necrosis factor, GO:0072584 caveolin-mediated endocytosis, GO:0090170 regulation of Golgi inheritance, GO:1900034 regulation of cellular response to heat, GO:1903351 cellular response to dopamine, GO:1904355 positive regulation of telomere capping, GO:1904417 positive regulation of xenophagy, GO:1905050 positive regulation of metallopeptidase activity, GO:2000641 regulation of early endosome to late endosome transport, GO:2000657 negative regulation of apolipoprotein binding
PPM1A	GO:0000287 magnesium ion binding F, GO:0003824 catalytic activity F, GO:0004721 phosphoprotein phosphatase activity F, GO:0004722 protein serine/threonine phosphatase activity F, GO:0004871 signal transducer activity F, GO:0005515 protein binding F, GO:0016787 hydrolase activity F, GO:0030145 manganese ion binding F, GO:0033192 calmodulin-dependent protein phosphatase activity F, GO:0043169 cation binding F, GO:0046872 metal ion binding F, GO:0070412 R-SMAD binding F, GO:0000122 negative regulation of transcription by RNA polymerase II P, GO:0006470 protein dephosphorylation P, GO:0006470 protein dephosphorylation P, GO:0006470 protein dephosphorylation P, GO:0006499 N-terminal protein myristoylation P, GO:0006499 N-terminal protein myristoylation P, GO:0007050 cell cycle arrest P, GO:0010991 negative regulation of SMAD protein complex assembly P, GO:0016055 Wnt signaling pathway P, GO:0016311 dephosphorylation P, GO:0030177 positive regulation of Wnt signaling pathway P, GO:0030512 negative regulation of transforming growth factor beta receptor signaling pathway P, GO:0030514 negative regulation of BMP signaling pathway P, GO:0035970 peptidyl-threonine dephosphorylation P, GO:0035970 peptidyl-threonine dephosphorylation P, GO:0042347 negative regulation of NF-kappaB import into nucleus P, GO:0043123 positive regulation of I-kappaB kinase/NF-kappaB signaling P, GO:0043124 negative regulation of I-kappaB kinase/NF-kappaB signaling P, GO:0045893 positive regulation of transcription, DNA-templated P, GO:0046827 positive regulation of protein export from nucleus P, GO:0071560 cellular response to transforming growth factor beta stimulus
PRG4	GO:0005044 scavenger receptor activity F, GO:0030247 polysaccharide binding F, GO:0006898 receptor-mediated endocytosis P, GO:0006955 immune response P, GO:0008283 cell proliferation

Disease Models & Mechanisms • Supplementary information

Name	Gene ontology annotation functions and processes
S100A12	GO:0005507 copper ion binding F, GO:0005509 calcium ion binding F, GO:0005515 protein binding F, GO:0008270 zinc ion binding F, GO:0046872 metal ion binding F, GO:0050786 RAGE receptor binding F,GO:0002376 immune system process P, GO:0002548 monocyte chemotaxis P, GO:0006805 xenobiotic metabolic process P, GO:0006954 inflammatory response P, GO:0030593 neutrophil chemotaxis P, GO:0031640 killing of cells of other organism P, GO:0042742 defense response to bacterium P, GO:0043123 positive regulation of I-kappaB kinase/NF-kappaB signaling P, GO:0043312 neutrophil degranulation P, GO:0043406 positive regulation of MAP kinase activity P, GO:0045087 innate immune response P, GO:0045576 mast cell activation P, GO:0050663 cytokine secretion P, GO:0050729 positive regulation of inflammatory response P, GO:0050832 defense response to fungus P, GO:0051092 positive regulation of NF-kappaB transcription factor activity P, GO:0061844 antimicrobial humoral immune response mediated by antimicrobial peptide P
S100A8	GO:0005509 calcium ion binding F, GO:0005515 protein binding, GO:0008017 microtubule binding, GO:0008270 zinc ion binding, GO:0035662 Toll-like receptor 4 binding, GO:0046872 metal ion binding, GO:0050544 arachidonic acid binding, GO:0050786 RAGE receptor binding, GO:0001816 cytokine production, GO:0002224 toll-like receptor signaling pathway P, GO:0002376 immune system process P, GO:0002523 leukocyte migration involved in inflammatory response P, GO:0002526 acute inflammatory response, GO:0002544 chronic inflammatory response, GO:0002793 positive regulation of peptide secretion, GO:0006914 autophagy, GO:0006915 apoptotic process, GO:0006919 activation of cysteine-type endopeptidase activity involved in apoptotic process, GO:0006935 chemotaxis, GO:0006954 inflammatory response, GO:0010043 response to zinc ion, GO:0014002 astrocyte development, GO:0018119 peptidyl-cysteine S-nitrosylation, GO:0019730 antimicrobial humoral response, GO:0030307 positive regulation of cell growth, GO:0030593 neutrophil chemotaxis, GO:0032119 sequestering of zinc ion, GO:0032496 response to lipopolysaccharide, GO:0032602 chemokine production, GO:0042060 wound healing, GO:0042742 defense response to bacterium, GO:0043312 neutrophil degranulation, GO:0045087 innate immune response, GO:0045087 innate immune response, GO:0045471 response to ethanol, GO:0050727 regulation of inflammatory response, GO:0050729 positive regulation of inflammatory response, GO:0050832 defense response to fungus, GO:0051092 positive regulation of NF-kappaB transcription factor activity, GO:0051493 regulation of cytoskeleton organization, GO:0070488 neutrophil aggregation, GO:2001244 positive regulation of intrinsic apoptotic signaling pathway

Disease Models & Mechanisms • Supplementary information

Name	Gene ontology annotation functions and processes
S100A9	GO:0004871 signal transducer activity, GO:0005509 calcium ion binding, GO:0005515 protein binding, GO:0008017 microtubule binding, GO:0008270 zinc ion binding, GO:0016209 antioxidant activity, GO:0035662 Toll-like receptor 4 binding, GO:0046872 metal ion binding, GO:0050544 arachidonic acid binding, GO:0050544 arachidonic acid binding, GO:0050786 RAGE receptor binding, GO:0050786 RAGE receptor binding, GO:0001816 cytokine production, GO:0002224 toll-like receptor signaling pathway, GO:0002376 immune system process, GO:0002523 leukocyte migration involved in inflammatory response, GO:0002793 positive regulation of peptide secretion, GO:0006417 regulation of translation, GO:0006914 autophagy, GO:0006914 autophagy, GO:0006915 apoptotic process, GO:0006919 activation of cysteine-type endopeptidase activity involved in apoptotic process, GO:0006935 chemotaxis, GO:0006954 inflammatory response, GO:0006954 inflammatory response, GO:0007267 cell-cell signaling, GO:0010976 positive regulation of neuron projection development, GO:0014002 astrocyte development, GO:0018119 peptidyl-cysteine S-nitrosylation, GO:0019730 antimicrobial humoral response, GO:0030194 positive regulation of blood coagulation, GO:0030307 positive regulation of cell growth, GO:0030593 neutrophil chemotaxis, GO:0030595 leukocyte chemotaxis, GO:0031532 actin cytoskeleton reorganization, GO:0032119 sequestering of zinc ion, GO:0032602 chemokine production, GO:0035606 peptidyl-cysteine S-trans-nitrosylation, GO:0035821 modification of morphology or physiology of other organism, GO:0042742 defense response to bacterium, GO:0043312 neutrophil degranulation, GO:0045087 innate immune response, GO:0045087 innate immune response, GO:0045113 regulation of integrin biosynthetic process, GO:0050727 regulation of inflammatory response, GO:0050729 positive regulation of inflammatory response, GO:0050832 defense response to fungus, GO:0051092 positive regulation of NF-kappaB transcription factor activity, GO:0051493 regulation of cytoskeleton organization, GO:0061844 antimicrobial humoral immune response mediated by antimicrobial peptide, GO:0070488 neutrophil aggregation, GO:0098869 cellular oxidant detoxification, GO:2001244 positive regulation of intrinsic apoptotic signaling pathway

Disease Models & Mechanisms • Supplementary information

Supplementary table 2a

Protein.ID	Protein.name	logFC	AveExpr	t	P.Value	q.Value	rank	lnOdds
W5QE95	Argonaute 1, RISC catalytic component	-22	-0.3	-35	1.22E-20	2.11E-16	1	26.6
W5PQA0	Beta-microseminoprotein	-19.1	-1	-12.4	2.57E-11	4.56E-09	98	14.1
W5NZV8	Angiopoietin like 7	-18.4	-2.3	-21.4	4.11E-16	1.19E-12	6	22.2
W5NQ88	Laminin subunit alpha 1	-17.8	-1.6	-17.1	4.39E-14	3.03E-11	25	19.5
ENSBTAP0 00000313 60	CD5 antigen-like	-17.6	-2.8	-17.6	2.40E-14	1.89E-11	22	19.9
A2P2H1	VH region (Fragment)	-16.5	-0.4	-15.7	2.40E-13	9.68E-11	42	17.8
W5P426	C-C motif chemokine ligand 14	-16.3	-1.3	-23.5	5.68E-17	2.87E-13	2	20.8
W5Q9W2	Uncharacterized protein	-16.1	-3.6	-20.4	1.14E-15	2.48E-12	8	20.5
W5PMH6	lipocalin 2	-16	-0.2	-19.6	2.43E-15	3.94E-12	10	21.4
W5NUP4	Amine oxidase	-15.9	-4.5	-11.2	1.80E-10	2.16E-08	145	13.1
W5P1J8	Amine oxidase (EC 1.4.3.-)	-15.8	0.8	-11	2.33E-10	2.72E-08	149	12.8
W5NWU4	Uncharacterized protein	-15.2	-4.9	-14.3	1.59E-12	4.69E-10	59	17
Q32PJ2	Apolipoprotein A-IV precursor	-13.9	2.4	-14.8	7.57E-13	2.43E-10	54	17.2
W5Q649	Breast cancer anti-estrogen resistance 3	-13.9	-5.9	-18	1.40E-14	1.28E-11	19	19.9
W5Q7I2	Uncharacterized protein (Fragment)	-13.9	3.3	-11.8	6.65E-11	1.02E-08	113	13.9
W5PGT6	Complement C6	-13.9	2.4	-13.9	2.67E-12	7.34E-10	63	16.4
W5PK55	Vitrin	-13.8	1.7	-12.5	2.11E-11	3.85E-09	95	14.8
W5PJ13	Pleiotrophin	-13.6	-6.7	-12.1	3.82E-11	6.36E-09	104	14.2
Q9XT27	Ceruloplasmin	-12.9	4.6	-12.8	1.35E-11	2.78E-09	84	15.1
W5Q5D7	Coiled-coil domain containing 18	-12.8	-5.4	-13.8	2.97E-12	7.93E-10	65	15.2
W5PNP1	Milk fat globule-EGF factor 8 protein	-12.4	5.2	-12.8	1.38E-11	2.82E-09	85	14.3
W5PCE0	Phospholipase B-like (EC 3.1.1.-)	-12.1	-5.2	-11.6	8.41E-11	1.22E-08	120	13
W5Q3F9	Aldehyde oxidase 1	-12	-7.1	-11.6	9.30E-11	1.26E-08	128	13.9
Q7M371	Plasma proteinase inhibitor (Fragment)	11.8	3.2	17.4	2.79E-14	2.11E-11	23	19.4
W5Q3Z7	Poly(U) binding splicing factor 60	11.8	-1.4	11.4	1.16E-10	1.47E-08	137	13.7
W5NUU7	Cadherin 5	11.6	1.1	13.1	7.87E-12	1.82E-09	75	15.8
W5P880	Proteoglycan 4	-11.6	5	-19.6	2.50E-15	3.94E-12	11	20.7
W5QHZ5	Uncharacterized protein	-11.4	3.1	-12.7	1.49E-11	2.95E-09	87	15.1
Q9MZW3	Type I collagen alpha 1 (Fragment)	11.3	-1.1	23.5	5.68E-17	2.87E-13	3	22.4
P02190	Myoglobin	-11.1	5.9	-16.9	5.10E-14	3.16E-11	28	18.9
W5P2Q0	Chondroadherin	-10.9	5.5	-13	9.77E-12	2.14E-09	79	14.5

Protein.ID	Protein.name	logFC	AveExpr	t	P.Value	q.Value	rank	lnOdds
W5PH95	Uncharacterized protein (Fragment)	-10.8	8.7	-17.6	2.36E-14	1.89E-11	21	19.6
W5PNK3	Aggrecan	-10.7	11.3	-16.2	1.21E-13	5.54E-11	38	18.1
W5P3G5	Profilin (Fragment)	10.7	-2	12.6	1.66E-11	3.16E-09	91	15.4
W5PDA9	C-type lectin domain family 3 member A	-10.4	3.7	-11.3	1.49E-10	1.82E-08	142	13
W5Q420	Reticulocalbin 1	10.2	1.6	12.9	1.09E-11	2.33E-09	81	14.4
P02083	Hemoglobin fetal subunit beta	9.9	7.3	11.9	5.29E-11	8.27E-09	111	13.4
W5PH81	Complement component C7 precursor	-9.8	2.9	-13.2	7.31E-12	1.71E-09	74	15.6
W5PQG6	Inositol-3-phosphate synthase 1	9.7	-2.9	12	4.69E-11	7.68E-09	106	14.1
W5PK03	Periostin	9.7	-2.3	18.4	9.77E-15	1.06E-11	16	19.8
W5PMS1	Cartilage oligomeric matrix protein	-9.6	12.3	-15.5	2.86E-13	1.13E-10	44	18
W5Q2F5	Decorin	-9.6	9.2	-17.7	2.14E-14	1.86E-11	20	19.7
W5PKS8	DEK proto-oncogene	9.4	-0.5	11.5	9.96E-11	1.30E-08	132	13.6
W5Q8V6	Cartilage intermediate layer protein	-9.3	9.9	-14.4	1.30E-12	4.03E-10	56	16.9
W5Q284	Hyaluronan and proteoglycan link protein 1	-9.3	7.1	-11.7	7.96E-11	1.18E-08	117	13.5
W5P7S6	Alpha-1-acid glycoprotein	-9.1	5.7	-13.6	3.93E-12	9.90E-10	68	16.1
W5P1I7	Ubiquitin conjugating enzyme E2 M	8.7	-0.8	11.1	2.03E-10	2.38E-08	148	13.2
ENSBTAP0 00000060 74	cartilage oligomeric matrix protein precursor	-8.7	11.1	-12.5	2.03E-11	3.75E-09	94	14.8
W5QAB1	Hemopexin	-8.6	7.9	-15.2	4.34E-13	1.60E-10	47	17
W5NU76	Fibromodulin (Fragment)	-8.5	8.9	-14.4	1.36E-12	4.15E-10	57	16.8
W5PEB0	Fatty acid binding protein 7	8.4	-2.9	19.7	2.41E-15	3.94E-12	9	20.8
B6E3I8	Enolase 1 (Fragment)	8.4	0.2	14.6	1.00E-12	3.16E-10	55	17.1
P21752	Thymosin beta-9	8.2	-0.1	13.4	5.54E-12	1.32E-09	73	15.7
W5QI48	Endosulfine alpha	8.2	-0.3	16.2	1.18E-13	5.54E-11	37	18.5
W5QE45	Serine/threonine-protein phosphatase (Fragment)	8.2	-2.1	13.4	5.25E-12	1.28E-09	71	15.8
W5PC55	Caspase 3	8.2	-3.1	22.5	1.40E-16	4.86E-13	5	22.2
Q8SQ25	Mannose-6-phosphate/insulin-like growth factor II receptor (Fragment) GN=m6p/igf2r PE=2 SV=1	7.9	-3.1	16.4	9.40E-14	5.26E-11	31	18.4
W5PJ95	Serine/threonine-protein phosphatase 2A 55 kDa regulatory subunit B	7.9	-3.1	18.2	1.11E-14	1.14E-11	17	19.9

Disease Models & Mechanisms • Supplementary information

Protein.ID	Protein.name	logFC	AveExpr	t	P.Value	q.Value	rank	lnOdds
W5P556	STE20 like kinase	7.8	-3.2	17.2	3.81E-14	2.76E-11	24	19.3
A4ZVY9	Beta-2-microglobulin	-7.7	3.9	-16.5	8.31E-14	4.80E-11	30	18.7
W5PBW8	Coatomer protein complex subunit zeta 2 (Fragment)	7.7	-3.2	21	6.21E-16	1.54E-12	7	21.6
W5QC84	Eukaryotic translation initiation factor 3 subunit D	7.7	-3.2	18.5	8.57E-15	9.91E-12	15	20
W5P7E2	Pyrophosphatase (inorganic) 1 (Fragment)	7.6	-3.2	16.1	1.35E-13	6.01E-11	39	18.3
W5PDR5	Complement C8 alpha chain	-7.6	4.9	-11.6	8.59E-11	1.22E-08	122	13.7
W5QH84	Transformer 2 beta homolog	7.5	-3.4	16.6	7.80E-14	4.67E-11	29	18.7
W5QAR2	Coagulation factor XIII A chain	-7.5	5.9	-23.4	6.61E-17	2.87E-13	4	22.9
W5Q0E7	Protein lin-7 homolog	7.5	-3.4	19.3	3.34E-15	4.14E-12	14	20.7
W5NS06	Annexin (Fragment)	-7.4	7.3	-12.6	1.74E-11	3.27E-09	92	14.7
Q9XSY9	Osteopontin	-7.4	4.9	-13.4	5.40E-12	1.30E-09	72	15.8
W5PZI1	Clusterin	-7.4	8.4	-19.4	3.09E-15	4.13E-12	13	18.8
W5PS39	KDEL motif containing 2	7.4	-3.6	17	4.72E-14	3.03E-11	27	18.7
W5Q2Y6	Rho-associated protein kinase (EC 2.7.11.1)	7.3	-3.5	18.1	1.29E-14	1.24E-11	18	19.7
W5QDG8	Fibronectin 1	-7.2	11.4	-19.5	2.84E-15	4.10E-12	12	20.4
C8BKE0	Cytoglobin	7.1	-3.6	16.4	9.71E-14	5.27E-11	32	18.6
W5P5P8	Proteasome assembly chaperone 3	7.1	-3.8	15.9	1.92E-13	8.11E-11	41	18.1
W5PEY2	Cathepsin V	7.1	-2.5	13.1	8.72E-12	1.97E-09	77	15.2
W5PTZ8	angiogenin-2-like	-7	6.6	-11	2.40E-10	2.77E-08	150	12.4
W5P7G7	Proteasome subunit beta type	7	-1	12.6	1.75E-11	3.27E-09	93	14.9
W5Q2B6	Epiphycan	-7	7.1	-11.9	5.03E-11	7.94E-09	110	13.8
W5P9G5	Vacuolar-sorting protein SNF8	7	-4.2	11.6	8.74E-11	1.22E-08	124	13.5
W5PCH3	Scinderin (Fragment)	-6.9	5.4	-15.7	2.40E-13	9.68E-11	43	17.8
W5P627	Gelsolin	-6.9	6.4	-11.6	9.01E-11	1.24E-08	126	13.5
W5NZ54	Replication protein A2	6.9	-3.7	16.3	1.15E-13	5.54E-11	35	18.4
W5PVD0	STIP1 homology and U-box containing protein 1	6.8	-4.1	11.6	8.73E-11	1.22E-08	123	13.9
ENSBTAP000000024466	immunoglobulin gamma 1 heavy chain constant region	-6.8	6.6	-12.2	3.08E-11	5.39E-09	99	14.2
W5QEH9	Thyroid hormone receptor associated protein 3	6.8	-3.6	15.1	5.13E-13	1.82E-10	49	17.4
D6PZY4	Factor H (Fragment)	-6.8	7.5	-13	9.27E-12	2.06E-09	78	15.2
W5NXW9	Immunoglobulin heavy constant mu (Fragment)	-6.7	6.7	-11.9	4.94E-11	7.87E-09	109	14.1
W5PGK9	TatD DNase domain containing 1 (Fragment)	6.7	-3.8	11.4	1.27E-10	1.59E-08	138	13.3

Protein.ID	Protein.name	logFC	AveExpr	t	P.Value	q.Value	rank	lnOdds
W5Q9D5	Vitronectin	-6.7	5.3	-11.2	1.68E-10	2.04E-08	143	13.2
W5PTS4	Ribonuclease A family member 4	-6.7	7.9	-16.3	1.13E-13	5.54E-11	34	18.2
W5PLB8	Erythrocyte membrane protein band 4.1 like 3	6.7	-3.9	14	2.19E-12	6.32E-10	60	16.1
W5Q7Z8	Small ubiquitin-related modifier (SUMO)	6.6	-3.7	16.3	1.08E-13	5.54E-11	33	18.6
W5NSL8	D-tyrosyl-tRNA(Tyr) deacylase	6.6	-3.9	13.7	3.31E-12	8.70E-10	66	16.1
W5QFP2	Histone cluster 1 H1 family member d	6.6	0.4	12.2	3.12E-11	5.41E-09	100	14.2
W5QB43	Cyclin dependent kinase 6	6.6	-3.8	13.6	4.15E-12	1.03E-09	70	15.9
W5PWN5	spermatid perinuclear RNA binding protein	6.6	-3.9	15	5.60E-13	1.91E-10	51	17.3
W5Q1W8	N-acetylglucosamine-1-phosphate transferase gamma subunit	6.6	-1.4	11.6	8.24E-11	1.20E-08	119	13.7
W5P4I5	Heterogeneous nuclear ribonucleoprotein H3	6.5	-3.8	14.3	1.52E-12	4.54E-10	58	15.8
W5QDT8	Tensin 1	6.5	-4.1	15.1	5.11E-13	1.82E-10	48	17.2
W5P4F0	Transcription factor BTF3	6.4	-3.9	15.1	5.40E-13	1.87E-10	50	17.5
W5P7U3	Sorbin and SH3 domain containing 1	6.4	-4	15.3	4.14E-13	1.56E-10	46	17.3
W5Q8N9	Ribosome biogenesis protein	6.4	-4	17	4.55E-14	3.03E-11	26	18.8
K4PF69	Ferritin	-6.4	5.8	-12.7	1.51E-11	2.95E-09	88	14.7
W5P8T5	Protein phosphatase, Mg2+/Mn2+ dependent 1B	6.3	-4	15.9	1.81E-13	7.86E-11	40	18
Q29543	Brain ribonuclease	-6.3	7.4	-13.7	3.71E-12	9.61E-10	67	16.1
W5PEX8	polycystin 1 like 2	6.3	-3.8	11.1	1.88E-10	2.24E-08	146	13
W5PG83	Serine/threonine-protein phosphatase 2A activator (EC 5.2.1.8) (Phosphotyrosyl phosphatase activator) (Fragment)	6.2	-1.6	11.8	6.46E-11	1.00E-08	112	13.9
W5QHL5	Calcium binding protein 39	6.2	-4.1	13.6	3.94E-12	9.90E-10	69	16
W5Q1B2	Splicing factor 3b subunit 1	6.1	-4	13.8	2.88E-12	7.81E-10	64	16.1
W5QG85	Obscurin like 1 (Fragment)	6.1	-4.2	15.3	3.97E-13	1.53E-10	45	17.6
W5NTC4	COMM domain containing 3	6.1	-4.3	12.4	2.43E-11	4.35E-09	97	14.5
Q2UVX4	Complement C3 precursor	-6	9.3	-11.6	9.13E-11	1.25E-08	127	13.6
W5NR63	Protein S100 (S100 calcium-binding protein)	-5.9	6.2	-11.7	6.96E-11	1.05E-08	115	13.6
W5QH50	Histidine rich glycoprotein	-5.9	6.5	-11.3	1.32E-10	1.63E-08	141	13.1
W5PJ45	Structural maintenance of chromosomes protein	5.9	-4.2	13	9.87E-12	2.14E-09	80	15.1
W5PUC1	Carbonic anhydrase 3	-5.9	7.7	-11.5	9.61E-11	1.28E-08	130	13.6
Q3SX28	Tropomyosin 2	5.7	-4.2	16.3	1.17E-13	5.54E-11	36	18.3

Protein.ID	Protein.name	logFC	AveExpr	t	P.Value	q.Value	rank	lnOdds
W5QFP0	Thrombospondin-1	-5.7	9.3	-11.4	1.11E-10	1.44E-08	134	13.3
B5AN56	Insulin-like growth factor-binding protein-6	-5.7	6.8	-11.5	9.98E-11	1.30E-08	133	13.5
W5QH35	Capping actin protein, gelsolin like	-5.7	5.4	-13.9	2.59E-12	7.29E-10	61	16.2
W5Q5Q6	Collagen type XIV alpha 1 chain	5.6	3.4	11.1	1.91E-10	2.26E-08	147	13.1
W5PFX1	Golgi associated PDZ and coiled-coil motif containing	5.6	-4.3	11.4	1.30E-10	1.61E-08	140	13.2
W5NRH2	complement C3-like	-5.6	6.9	-12.7	1.64E-11	3.16E-09	90	14.9
W5P8E3	Fibroblast activation protein alpha	5.6	-4.1	11.5	9.63E-11	1.28E-08	131	13.4
W5QI29	Extracellular matrix protein 1	-5.6	5.5	-14.9	7.12E-13	2.33E-10	53	17
P12303	Transthyretin	-5.6	8.5	-14.9	6.66E-13	2.22E-10	52	17.1
W5PIG7	Enolase 1	-5.5	11	-12.9	1.21E-11	2.54E-09	82	15.1
P09670	Superoxide dismutase [Cu-Zn]	-5.5	7.1	-12	4.90E-11	7.87E-09	107	14.1
W5Q9Z3	ABI family member 3 binding protein	-5.3	6.6	-11.7	7.15E-11	1.07E-08	116	13.5
W5NY95	complement factor B	-5.3	9.2	-11.4	1.28E-10	1.59E-08	139	13.3
W5P5W9	Triosephosphate isomerase (Fragment)	-5.1	8.6	-11.2	1.77E-10	2.13E-08	144	13.1
D8X187	Serpin peptidase inhibitor clade B ovalbumin member 1	-5.1	5.9	-13.1	8.13E-12	1.86E-09	76	15.2
W5Q5G8	Transketolase	-5	6.9	-12.9	1.22E-11	2.54E-09	83	15.1
W5PJ66	Complement factor D	-5	6.5	-12.2	3.56E-11	6.06E-09	102	14
M4T8F1	Peroxiredoxin 6	-5	7.7	-12.7	1.51E-11	2.95E-09	89	15.1
W5P1X9	Fructose-bisphosphate aldolase (EC 4.1.2.13)	-4.9	9.2	-11.4	1.13E-10	1.44E-08	135	13.5
F2YQ13	Gelsolin isoform b	-4.9	10.6	-12.2	3.21E-11	5.52E-09	101	14.3
W5QC41	Pyruvate kinase (Fragment)	-4.9	9.4	-11.6	8.56E-11	1.22E-08	121	13.7
W5PDP6	Complement C1q B chain	-4.8	3.4	-11.7	6.87E-11	1.05E-08	114	13.9
Q5MIB5	Glycogen phosphorylase, liver form	-4.8	5.3	-13.9	2.61E-12	7.29E-10	62	16.3
W5P8I3	ribonuclease UK114	-4.8	5.1	-12.7	1.49E-11	2.95E-09	86	14.8
W5Q2E1	Lumican	-4.7	8.4	-12.4	2.31E-11	4.18E-09	96	14.7
W5PP64	four and a half LIM domains 1 (Fragment)	-4.5	7.6	-11.6	8.90E-11	1.24E-08	125	13.7
Q7M2U8	Apolipoprotein E	-4.5	4.9	-11.9	4.92E-11	7.87E-09	108	14.2
W5PG95	heat shock 70kDa protein 1A	-4.4	8	-11.5	9.60E-11	1.28E-08	129	13.6
W5P8W6	Collagen type XII alpha 1 chain	-4.4	9.3	-12.1	3.88E-11	6.42E-09	105	14.3
W5PK04	Phosphoglycerate mutase (EC 5.4.2.11) (EC 5.4.2.4)	-4.2	8.2	-11.6	8.18E-11	1.20E-08	118	13.5

Protein.ID	Protein.name	logFC	AveExpr	t	P.Value	q.Value	rank	lnOdds
W5PPG3	Aldehyde dehydrogenase 9 family member A1 (Fragment)	-4.1	5.9	-11.4	1.13E-10	1.44E-08	136	13.5
W5NYH2	Nucleoside diphosphate kinase (Fragment)	-3.9	7.7	-12.1	3.81E-11	6.36E-09	103	14.3

Supplementary table 2b

Protein.ID	Protein.name	logFC	AveExpr	t	P.Value	q.Value	rank	lnOdds
W5Q8U5	Chordin like 1	-14.2	-5.7	-8.5	2.43E-08	1.91E-05	22	8.9
W5QA07	protein HP-25 homolog 1	-13.5	0.5	-10.2	8.73E-10	1.26E-06	12	12.2
W5PM87	mitogen-activated protein kinase kinase 2	-13.1	-6.7	-11.1	1.89E-10	2.97E-07	11	13.6
W5NS08	TNF alpha induced protein 8 like 3	-13	-5.8	-7.8	1.02E-07	6.32E-05	28	7.8
W5P7Y9	Class II major histocompatibility complex transactivator	-12.6	-6.9	-8.2	4.10E-08	3.09E-05	23	8.7
W5QC30	Tubulin alpha chain (Fragment)	-12.6	-5.1	-7.2	3.78E-07	0.0002	32	6.6
W5Q983	Glycerol-3-phosphate dehydrogenase [NAD(+)] (EC 1.1.1.8)	11.9	-4	7.1	4.38E-07	0.00022	33	6.5
W5PES7	Maturin, neural progenitor differentiation regulator homolog (Fragment)	-11.9	-5.6	-13.8	2.85E-12	1.65E-08	3	16.8
W5QH23	Mannan binding lectin serine peptidase 1	11.1	-4.5	8.1	5.34E-08	3.86E-05	24	8.4
W5PGP8	Queuosine salvage protein	-10.5	-6.1	-7	4.90E-07	0.00024	35	6.4
W5NWL0	Coiled-coil domain containing 88A	-10.5	-4.8	-5.4	1.88E-05	0.00562	58	3
W5QB02	Transglutaminase 2 (Fragment)	10.4	-4.7	5.4	2.11E-05	0.00622	59	2.9
W5Q545	TGF-beta activated kinase 1 (MAP3K7) binding protein 1 (Fragment)	10.3	-6.6	7.1	4.41E-07	0.00022	34	6.4
W5QF93	BCL2 like 15	10.3	-4.4	7.7	1.11E-07	6.67E-05	29	7.7
W5PZT2	Angiopoietin like 6	-10.3	-6	-12.5	1.96E-11	6.80E-08	5	14.6
W5PCD9	Echinoderm microtubule associated protein like 4	10.2	-6.2	8.7	1.66E-08	1.44E-05	20	9.2
P50415	Cathelicidin-3	10.2	-3.4	7.6	1.37E-07	7.94E-05	30	7.5
W5Q1I8	Protein phosphatase, Mg2+/Mn2+ dependent 1F (Fragment)	-10.1	-6	-10	1.32E-09	1.63E-06	14	11.6
Q9UE12	Keratin, type I cuticularÂ	10.1	-6.5	5.4	2.21E-05	0.00638	60	2.8
W5PQA0	Beta-microseminoprotein	9.9	-1	6.5	1.73E-06	0.00075	40	5.1
W5P1T4	Serine/threonine-protein phosphatase	9.9	-6.2	11.1	1.86E-10	2.97E-07	10	12.9
P79362	Cathelicidin-2	9.8	-1.1	5.8	8.80E-06	0.00294	52	3.6
W5Q438	G protein subunit beta 2	-9.6	-5.3	-7.5	1.64E-07	9.20E-05	31	7
W5PFT7	Fructose-bisphosphatase 2	9.6	-3	6.1	3.67E-06	0.00133	48	4.5
W5PQQ6	NECAP endocytosis associated 2	-9.5	-7	-11.5	9.55E-11	2.07E-07	8	13.4

Disease Models & Mechanisms • Supplementary information

Protein.ID	Protein.name	logFC	AveExpr	t	P.Value	q.Value	rank	lnOdds
W5PXE3	Adhesion regulating molecule 1	-9.3	-5.7	-4.8	9.65E-05	0.02462	68	1.5
W5P2N6	Adenosylhomocysteinase	-9.3	-7	-6.6	1.37E-06	0.00061	39	5.4
W5P9L9	heterogeneous nuclear ribonucleoprotein K	-9.2	-5.9	-5.5	1.81E-05	0.00552	57	2.9
W5PBW1	Serine dehydratase (Fragment)	8.9	-2.4	9.4	3.89E-09	4.22E-06	16	10.3
W5PDL7	Polypeptide N-acetylgalactosaminyltransferase (EC 2.4.1.-) (Protein-UDP acetylgalactosaminyltransferase)	8.9	-5.8	14.5	1.18E-12	1.65E-08	1	16.2
W5PWZ2	Galectin (Fragment)	-8.9	-5.5	-11.4	1.14E-10	2.20E-07	9	12.8
W5QJ04	Protein phosphatase, Mg2+/Mn2+ dependent 1A	8.9	-5	6.1	3.91E-06	0.00136	49	4.4
W5QHP4	Actin like 6A (Fragment)	-8.9	-6.9	-7	5.06E-07	0.00024	36	6.2
Q1RMN8	Immunoglobulin light chain, lambda gene cluster	8.9	-3	5.6	1.20E-05	0.00393	53	3.4
W5QDK7	X-ray repair cross complementing 5	-8.9	-5	-6.1	3.93E-06	0.00136	50	4.4
W5P4Q1	Transportin 1	8.9	-5.9	5.3	2.43E-05	0.00691	61	2.7
W5NWF7	Elongation factor Tu (Fragment)	8.8	-5.6	6.2	3.41E-06	0.00129	46	4.5
W5PMH6	LCN2 lipocalin 2	8.7	-0.2	10	1.26E-09	1.63E-06	13	11.5
M4WED3	Cell division cycle 42	8.7	-6.4	4.5	0.00018	0.04268	73	0.9
W5NUR8	Rabaptin, RAB GTPase binding effector protein 2 (Fragment)	-8.7	-6.3	-7.8	9.25E-08	5.94E-05	27	7.9
W5PB83	Serine hydroxymethyltransferase	8.6	-6.1	6.6	1.22E-06	0.00056	38	5.4
W5PK29	Striatin	8.5	-5.1	12.3	2.70E-11	7.82E-08	6	13.9
W5PRS4	Peptidylprolyl isomerase (EC 5.2.1.8)	-8.5	-6.8	-6.3	2.79E-06	0.00112	43	4.7
W5NQJ0	Protein S100 (S100 calcium-binding protein)	8.4	-2.5	7.9	7.48E-08	4.99E-05	26	7.8
W5PY97	Supervillin	-8.3	-6.4	-8	6.52E-08	4.53E-05	25	7.6
Q30B83	Ribosomal protein L35a (Fragment)	8.2	-6	5.3	2.88E-05	0.00807	62	2.5
O62804	Transcription factor GABP alpha subunit (Fragment)	8.2	-6.5	8.6	1.84E-08	1.52E-05	21	8.8
W5PB46	Phospholipid transfer protein	-8.2	-4.5	-4.6	0.00014	0.03488	70	1.1
W5Q149	Oxidation resistance 1	-8.2	-6.1	-13.9	2.80E-12	1.65E-08	2	15.8
W5QIN8	Fibrillin 1	8.1	-5.1	5.5	1.53E-05	0.00474	56	3.1
W5PG09	Phosphoglycerate kinase	-8.1	-6.5	-8.8	1.30E-08	1.25E-05	18	9.1
W5Q813	NOP58 ribonucleoprotein	8.1	-6	4.7	0.00011	0.02868	69	1.3

Protein.ID	Protein.name	logFC	AveExpr	t	P.Value	q.Value	rank	lnOdds
W5P8Z5	myosin-13	7.9	-7	8.7	1.48E-08	1.35E-05	19	8.9
W5PTH6	Amidophosphoribosyltransferase	7.5	-6.8	8.9	1.05E-08	1.07E-05	17	9.2
W5PS78	opioid growth factor receptor (Fragment)	-7.5	-6.8	-9.9	1.58E-09	1.83E-06	15	11.2
W5P748	Galectin	-7.5	-7	-6	5.42E-06	0.00184	51	4.2
W5NQK9	S100 calcium binding protein A8 (Fragment)	7.5	-2	6.2	3.50E-06	0.00129	47	4.5
W5PCE0	Phospholipase B-like (EC 3.1.1.-)	7.4	-5.2	6.7	1.04E-06	0.00049	37	5.4
W5PK00	Heterogeneous nuclear ribonucleoprotein H2	-7.2	-7.1	-6.3	2.51E-06	0.00104	42	4.6
A2P2H1	VH region (Fragment)	7.2	-0.4	6.2	3.06E-06	0.00118	45	4.5
W5Q399	Ubiquitin conjugating enzyme E2 L3	7.1	-6.9	5.6	1.26E-05	0.00402	54	3.3
W5Q8P8	MYC binding protein	6.9	-6.4	4.9	6.36E-05	0.01725	64	1.8
W5QD75	G protein subunit alpha i3 (Fragment)	-6.9	-6.3	-13.5	4.69E-12	2.03E-08	4	15.4
W5Q9P0	Catenin beta like 1	-6.6	-6.5	-11.8	6.42E-11	1.59E-07	7	13.6
ENSBTAP00000024146	Alpha-2-macroglobulin isoform X4	-6.3	-5.6	-6.2	3.06E-06	0.00118	44	4.6
W5QGZ4	Prolyl 3-hydroxylase 2	-4.3	-7.8	-4.6	0.00016	0.03837	71	1
W5P9P1	Actinin alpha 3 (gene/pseudogene)	-4.2	-7.8	-6.5	1.78E-06	0.00075	41	5.1
W5P880	Proteoglycan 4	3.2	5	5.1	4.62E-05	0.01272	63	2.1
W5PTL2	Complement factor properdin	2.5	4.2	4.5	0.0002	0.04762	74	0.8
W5PDP6	Complement C1q B chain (Fragment)	2.4	3.4	5.6	1.27E-05	0.00402	55	3.3
W5QGB5	Histone H4 (Fragment)	2.2	5.2	4.6	0.00016	0.03837	72	1
W5PDQ9	Complement C1q C chain	2	4.4	4.9	7.77E-05	0.02042	66	1.7
W5QI29	Extracellular matrix protein 1	1.7	5.5	4.8	9.12E-05	0.02363	67	1.5
W5QDG8	Fibronectin 1	1.7	11.4	4.9	7.11E-05	0.01897	65	1.7

Supplementary table 2c

Protein.ID	Protein.name	logFC	AveExpr	t	P.Value	q.Value	rank	lnOdds
W5PQA0	Beta-microseminoprotein	-18.3	-1	-13.6	4.16E-12	1.64E-09	44	16.1
P54230	Cathelicidin-1	18.2	-0.4	12.9	1.11E-11	3.36E-09	56	15.4
Q7M371	SHEEP Plasma proteinase inhibitor (Fragment)	18.2	3.2	27.1	2.83E-18	2.45E-14	2	24.4
P79362	Cathelicidin-2	17.8	-1.1	13.1	7.82E-12	2.66E-09	51	15.8
W5NW78	resistin	16.8	-3.4	10.1	1.04E-09	1.54E-07	117	11.8
W5PSC1	Uncharacterized protein (Fragment)	16.4	-3.7	11.6	8.43E-11	1.85E-08	79	13.7
W5QFM1	Histone H2A	16.3	1	13.5	4.92E-12	1.86E-09	46	16.1
W5PKA9	Coagulation factor V	15.9	1.9	12.2	3.29E-11	8.66E-09	66	14.6
W5NQK9	S100 calcium binding protein A8 (Fragment)	15.8	-2	14.3	1.61E-12	7.15E-10	39	16.7
W5NVG2	EGF containing fibulin like extracellular matrix protein 1	15.7	1.3	11.2	1.65E-10	3.25E-08	88	13.4
P50415	Cathelicidin-3	15.5	-3.4	10.5	5.51E-10	9.19E-08	104	12.2
W5NQH6	Protein S100 (S100 calcium-binding protein)	15.4	-2	10.5	5.69E-10	9.32E-08	106	12.2
W5QFP2	Histone cluster 1 H1 family member d	15.2	0.4	30.3	2.63E-19	4.56E-15	1	25.3
W5QI04	Tropomyosin alpha-1 chain (Fragment)	15.2	3	9	9.52E-09	1.12E-06	147	9.9
W5NS65	histone H1.4-like	15.1	-1	10.7	3.84E-10	6.87E-08	97	12.6
W5PT14	Importin 7	15.1	-2	8.9	9.92E-09	1.15E-06	149	10
W5Q5L0	Eukaryotic translation initiation factor 4B	-15.1	0.8	-9.3	4.48E-09	5.67E-07	137	10.6
W5Q0L1	Eukaryotic translation elongation factor 1 gamma	-14.8	0.9	-19.1	4.25E-15	5.68E-12	13	21.6
W5PV90	Eukaryotic translation initiation factor 5B	14.8	-0.3	10.8	3.44E-10	6.29E-08	95	12.7
W5PYG7	Eukaryotic translation initiation factor 3 subunit L (Fragment)	-14.6	-0.4	-11.3	1.49E-10	3.03E-08	85	13.6
W5Q8R4	DNA helicase (EC 3.6.4.12)	14.6	-1.6	10.1	1.22E-09	1.76E-07	120	11.8
W5Q7R4	Inhibin beta A chain	14.6	-0.9	13	1.00E-11	3.22E-09	53	15.6
Q1RMN8	Immunoglobulin lambda-like polypeptide 1	14.6	-3	9.5	3.07E-09	4.06E-07	131	10.8
W5QEG5	SPT16 homolog, facilitates chromatin remodeling subunit	-14.4	0.3	-9.4	4.05E-09	5.21E-07	135	10.7
W5PMQ9	SUMO1 activating enzyme subunit 1	-14.4	0.9	-9.6	2.73E-09	3.65E-07	129	11.2
A2P2G9	VH region (Fragment)	14.4	-4	12.9	1.11E-11	3.36E-09	57	15.3
W5Q6B3	Prefoldin subunit 4	-14.3	0.1	-9.6	2.73E-09	3.65E-07	130	11
W5PA79	Splicing factor 3a subunit 1	14.3	-0.5	10.6	4.75E-10	8.24E-08	100	12.7

Protein.ID	Protein.name	logFC	AveExpr	t	P.Value	q.Value	rank	lnOdds
W5PTW0	G protein nucleolar 1 (putative)	-14	-0.9	-13.4	5.21E-12	1.92E-09	47	15.7
P08779	Keratin, type I cytoskeletal 16	13.9	0	9.4	4.42E-09	5.64E-07	136	10.6
W5PXI2	HDGF like 3 (Fragment)	-13.9	-2	-9.8	2.10E-09	2.99E-07	122	11.4
W5QCY6	GMP reductase (Fragment) OS	-13.8	0.2	-19.6	2.47E-15	4.28E-12	10	21.6
W5PXR1	Ectonucleotide pyrophosphatase/phosphodie sterase 1 (Fragment)	-13.8	-0.7	-11.6	8.67E-11	1.88E-08	80	14.3
P49929	Cathelin-related peptide SC5	13.8	-3.6	11.5	9.44E-11	2.02E-08	81	13.3
W5NY01	scavenger receptor cysteine-rich type 1 protein M130	13.7	0.3	9.5	3.38E-09	4.41E-07	133	10.8
W5NY53	Mannose-6-phosphate isomerase	-13.7	-1.5	-9.1	7.82E-09	9.42E-07	144	10.2
W5NWP4	DEAD-box helicase 3, X-linked	-13.6	-0.2	-16.3	1.04E-13	8.79E-11	20	19
W5NR38	RB binding protein 9, serine hydrolase	13.6	-0.8	10.5	5.47E-10	9.19E-08	103	12.6
W5NYH4	BCL2 associated athanogene 3	-13.6	-1.4	-11.7	7.95E-11	1.77E-08	78	14.1
W5NQJ0	Protein S100 (S100 calcium-binding protein)	13.5	-2.5	14.2	1.70E-12	7.37E-10	40	16.7
W5Q5R5	SAM and HD domain containing deoxynucleoside triphosphate triphosphohydrolase 1	13.4	-0.7	14.3	1.45E-12	6.82E-10	37	16.8
W5PZ86	Septin 8	-13.3	0.8	-12.2	3.58E-11	9.27E-09	67	14.6
ENSBTAP0 00000230 55	Hepatocyte growth factor activator preproprotein	13.3	0	10.3	7.32E-10	1.15E-07	110	12.2
W5Q0C0	Procollagen-lysine,2-oxoglutarate 5-dioxygenase 3	13.3	0.6	10.1	1.04E-09	1.54E-07	116	11.8
W5Q7V5	Immunoglobulin superfamily containing leucine rich repeat	-13.2	0.1	-15.8	1.97E-13	1.40E-10	24	18.7
W5Q831	Destrin, actin depolymerizing factor	-13.2	1.4	-12	4.36E-11	1.08E-08	70	14.1
W5PKP3	Family with sequence similarity 114 member A1	-13.2	0.9	-11.4	1.23E-10	2.53E-08	84	13.7
W5P2U4	eukaryotic peptide chain release factor subunit 1	13.1	-0.7	10.3	7.42E-10	1.16E-07	111	12.3
W5QFK2	Microtubule-actin crosslinking factor 1	13.1	-1.3	12.8	1.28E-11	3.71E-09	60	15.7
W5PJG0	Serum amyloid A protein	13.1	-5.8	10.7	3.61E-10	6.52E-08	96	12.5
B5AZT5	Insulin-like growth factor binding protein-4	13.1	-0.4	11.5	1.08E-10	2.29E-08	82	13.2

Protein.ID	Protein.name	logFC	AveExpr	t	P.Value	q.Value	rank	lnOdds
W5NQK6	LIM and senescent cell antigen-like-containing domain protein	-13	0.4	-21.1	5.35E-16	1.55E-12	6	22.6
W5PKQ1	Sorbin and SH3 domain containing 3	-13	-1.1	-15.7	2.29E-13	1.40E-10	27	19
W5P4S2	Thioredoxin like 1	-12.9	-0.2	-15.8	2.11E-13	1.40E-10	26	18.6
W5QED7	ADP-ribosylhydrolase like 2	-12.9	0.3	-16.8	5.92E-14	5.70E-11	18	19.4
W5Q3G5	Small muscle protein, X-linked	-12.8	-1.9	-10.4	7.03E-10	1.12E-07	109	12.2
W5NTV7	Endothelial differentiation related factor 1 (Fragment)	-12.7	-0.3	-15.1	5.38E-13	2.83E-10	33	17.9
W5PBW1	Serine dehydratase	12.6	-2.4	14.5	1.12E-12	5.42E-10	36	17
W5PPC2	Heterogeneous nuclear ribonucleoprotein D like	-12.6	-0.6	-14.8	7.55E-13	3.74E-10	35	17.6
W5PKQ5	Microtubule associated protein 1A	12.5	-2	11.1	1.89E-10	3.69E-08	89	13.4
W5PG20	Secernin 1 (Fragment)	-12.5	-0.5	-15.6	2.62E-13	1.52E-10	30	18.4
W5PS11	Phosphoserine aminotransferase (Fragment)	12.5	-1.1	9.1	7.07E-09	8.64E-07	142	10.2
W5Q106	PDZ and LIM domain 4 (Fragment)	-12.4	-1.8	-11	2.34E-10	4.47E-08	91	13.1
W5P7W2	Stromal cell derived factor 4 (Fragment)	-12.4	-0.9	-19.7	2.21E-15	4.25E-12	9	21.6
W5NQM8	Cortactin (Fragment)	12.4	0.3	10.3	7.55E-10	1.16E-07	112	12.3
W5Q0A8	Glucosamine-6-phosphate isomerase (EC 3.5.99.6) (Glucosamine-6-phosphate deaminase)	-12.3	-0.9	-12.4	2.27E-11	6.06E-09	65	15.1
W5Q5C7	Catenin alpha 1	-12.3	-1.2	-13	1.04E-11	3.27E-09	55	15.8
W5PLE0	doublecortin like kinase 1 (Fragment)	12.2	-1.6	9.1	7.21E-09	8.75E-07	143	10.2
W5NQ92	elongin B (Fragment)	-12.2	0.2	-18.6	7.30E-15	9.04E-12	14	20.3
B3GS77	Insulin-like growth factor binding protein 5	12.2	2	13.3	5.90E-12	2.13E-09	48	14.9
W5Q6V5	Dynamin 2	12.1	-0.7	9.4	4.02E-09	5.20E-07	134	10.8
W5NUI6	N-sulfoglucosamine sulfohydrolase	-12.1	-2.8	-10.5	6.00E-10	9.63E-08	108	12.5
W5PIE3	Protein phosphatase methylesterase 1 (PME-1) (EC 3.1.1.-) (Fragment)	12.1	-2	9.3	4.88E-09	6.09E-07	139	10.6
W5PHQ4	Nudix hydrolase 2	-12.1	-1	-14.2	1.83E-12	7.75E-10	41	16.6
W5P0N8	Proteasome activator subunit 3	-12.1	-0.6	-21.4	4.16E-16	1.44E-12	5	22

Disease Models & Mechanisms • Supplementary information

Protein.ID	Protein.name	logFC	AveExpr	t	P.Value	q.Value	rank	lnOdds
W5QCR6	Microtubule-associated protein	12.1	-1.7	13	1.00E-11	3.22E-09	54	15.5
W5PVU5	Proteasome subunit beta type	12	-1.4	10.5	5.86E-10	9.50E-08	107	12.4
W5PZ59	Histone H2A	12	-1.7	11	2.24E-10	4.32E-08	90	13.2
P21752	Thymosin beta-9	12	-0.1	20	1.63E-15	3.54E-12	8	21.2
W5NRM7	Cellular retinoic acid binding protein 1	-12	2.7	-11.7	7.35E-11	1.66E-08	77	13.5
W5PK92	Lysyl oxidase like 2 (Fragment)	11.9	0.7	10.1	1.10E-09	1.61E-07	119	11.5
W5P181	Nuclear distribution C, dynein complex regulator	-11.9	-1.3	-10.3	7.59E-10	1.16E-07	113	12.2
W5PY64	H1 histone family member X (Fragment)	11.9	-1.2	10.5	5.56E-10	9.19E-08	105	12.4
W5NSN2	Collagen type XVI alpha 1 chain	11.9	1.4	10.6	4.68E-10	8.20E-08	99	12.5
W5Q649	Breast cancer anti-estrogen resistance 3	-11.8	-5.9	-16.4	9.57E-14	8.74E-11	19	19.1
W5PV54	Carboxypeptidase N subunit 1	11.8	-1.2	10	1.23E-09	1.76E-07	121	11.6
B6E3I8	Enolase 1 (Fragment)	11.7	0.2	20.5	1.01E-15	2.51E-12	7	21.5
W5NUU7	Cadherin 5	11.7	1.1	13.3	6.57E-12	2.33E-09	49	16
W5PLS7	Growth factor receptor bound protein 2	11.7	-0.4	15.5	3.20E-13	1.79E-10	31	18.1
W5PJ59	SH3 domain binding glutamate rich protein	-11.6	-1.2	-15.7	2.33E-13	1.40E-10	28	18.5
W5PG42	high mobility group box 3	-11.6	-1.4	-16.3	1.06E-13	8.79E-11	21	19.2
C9E8M7	Cytochrome b5	11.5	-0.3	9.5	3.12E-09	4.10E-07	132	10.5
P50122	Metalloproteinase inhibitor 1	11.5	-0.1	9.7	2.37E-09	3.30E-07	124	10.8
W5NZ17	Cysteine and histidine rich domain containing 1 (Fragment)	11.5	-0.2	10.2	9.45E-10	1.43E-07	115	12
W5PSG8	HD domain containing 3	-11.3	-1.3	-15.3	3.72E-13	2.02E-10	32	17.8
W5Q3D2	Sorting nexin 12	-11.2	0.6	-15	5.78E-13	2.95E-10	34	16.9
W5QCQ3	Elastin microfibril interfacer 1	11.2	-0.7	8.9	9.87E-09	1.15E-06	148	10
W5QGL6	Non-specific serine/threonine protein kinase (EC 2.7.11.1)	11.1	-0.4	12.8	1.34E-11	3.77E-09	61	15.4
W5PHQ0	Myeloperoxidase (Fragment)	11.1	-5	15.8	2.02E-13	1.40E-10	25	18.2
W5PEN2	Tumor protein D52	-11.1	0.3	-17.7	2.06E-14	2.24E-11	16	18.9
W5NPC7	Acyl-CoA binding domain containing 3 (Fragment)	-11	0.9	-13.9	2.50E-12	1.03E-09	42	15.8
W5PME6	Zinc finger CCCH-type containing, antiviral 1 like	-11	-0.2	-11.4	1.17E-10	2.45E-08	83	13.8
W5QAP3	Target of myb1 membrane trafficking protein (Fragment)	11	-0.6	14.3	1.52E-12	6.94E-10	38	16.9

Protein.ID	Protein.name	logFC	AveExpr	t	P.Value	q.Value	rank	lnOdds
W5PP47	Cystatin-B (Fragment)	11	-1.8	9	8.61E-09	1.02E-06	146	9.8
W5PSB5	GDP-mannose pyrophosphorylase B	-10.9	-1.4	-16.1	1.48E-13	1.15E-10	22	16.7
W5NY25	Eukaryotic translation initiation factor 3 subunit C	10.9	0.1	10.9	2.91E-10	5.38E-08	94	12.9
W5NSD5	RAP1B, member of RAS oncogene family	10.9	-0.6	9.2	5.57E-09	6.86E-07	141	10.2
W5PHU8	aminoacyl tRNA synthase complex-interacting multifunctional protein 1	10.9	-0.9	9.7	2.38E-09	3.30E-07	125	11.1
W5PI22	canopy FGF signaling regulator 2	-10.8	1.9	-12.1	4.16E-11	1.05E-08	69	13.9
W5PMG4	PYM homolog 1, exon junction complex associated factor	-10.8	-1.4	-12.9	1.12E-11	3.36E-09	58	15.1
W5QHV6	Sorting nexin 1	-10.8	0.8	-12.6	1.92E-11	5.20E-09	64	14.5
W5QFC2	Four and a half LIM domains 3	10.7	-1.9	11.8	5.92E-11	1.39E-08	73	14.3
W5Q4B7	Rap1 GTPase-GDP dissociation stimulator 1	10.6	-2.8	9.7	2.40E-09	3.31E-07	126	11.1
W5Q9C4	Nitrilase family member 2	-10.6	-1.1	-24.6	2.18E-17	1.26E-13	3	23.2
W5PP53	Cold inducible RNA binding protein	-10.6	-1.2	-12.1	4.16E-11	1.05E-08	68	14.5
W5PVZ9	Dystroglycan 1	10.5	0.7	9.3	5.16E-09	6.40E-07	140	10.1
W5NUZ1	Dynein cytoplasmic 1 intermediate chain 2	-10.5	-0.9	-22.8	1.12E-16	4.84E-13	4	22.7
W5QDU4	Tetratricopeptide repeat domain 38 (Fragment)	-10.5	-2.9	-12	4.41E-11	1.08E-08	71	14.3
W5PTM9	ubiquitin conjugating enzyme E2 V2 (Fragment)	10.5	-0.8	12	4.86E-11	1.17E-08	72	14.4
W5PUT8	Actin-related protein 2/3 complex subunit 5	10.4	-1.4	8.9	1.10E-08	1.27E-06	150	9.6
W5PPQ6	SEC13 homolog, nuclear pore and COPII coat complex component	-10.3	0.1	-15.7	2.34E-13	1.40E-10	29	17.5
W5PDD6	Secernin 2 (Fragment)	-10.3	-2	-13.5	4.51E-12	1.74E-09	45	15.8
W5PR73	Coronin (Fragment)	-10.3	0.2	-10.6	4.39E-10	7.78E-08	98	12.1
W5PXZ3	alpha-fetoprotein-like	10.3	-1.3	9.3	4.88E-09	6.09E-07	138	10.4
W5PZ50	TBC1 domain family member 15 (Fragment)	10.1	-0.3	10.9	2.57E-10	4.85E-08	92	12.5
W5PPZ9	guanidinoacetate N-methyltransferase (Fragment)	10.1	-1.8	11.8	6.01E-11	1.39E-08	75	14.2
W5P7G7	Proteasome subunit beta type	10.1	-1	18.2	1.14E-14	1.32E-11	15	20.1

Disease Models & Mechanisms • Supplementary information

Protein.ID	Protein.name	logFC	AveExpr	t	P.Value	q.Value	rank	lnOdds
W5P8I5	elongin C	-10	0.9	-10.3	8.01E-10	1.22E-07	114	11.6
W5PKS8	DEK proto-oncogene	10	-0.5	13.2	6.84E-12	2.37E-09	50	16
W5P1I7	Ubiquitin conjugating enzyme E2 M (Fragment)	10	-0.8	12.8	1.24E-11	3.65E-09	59	15.5
W5PV21	Translin	9.9	-1.9	12.6	1.83E-11	5.04E-09	63	15
W5QI48	Endosulfine alpha	9.8	-0.3	19.5	2.71E-15	4.28E-12	11	20.9
W5Q8X3	Metallo-beta-lactamase domain containing 1 (Fragment)	-9.8	-3	-19.3	3.65E-15	5.27E-12	12	20.7
W5Q3F9	Aldehyde oxidase 1	-9.7	-7.1	-10.1	1.06E-09	1.56E-07	118	12
W5P6P2	ribosomal protein L7	9.7	-1.2	9.6	2.61E-09	3.53E-07	128	10.4
W5NS79	bifunctional 3'-phosphoadenosine 5'-phosphosulfate synthase 2-like	-9.6	-2	-11.2	1.63E-10	3.25E-08	86	12.8
C5IWV3	Protein disulfide isomerase family A member 5	9.6	-0.8	13.9	2.78E-12	1.12E-09	43	16.3
W5P6V4	Golgi glycoprotein 1	-9.5	-1.2	-11.2	1.64E-10	3.25E-08	87	12.5
W5QE39	Ribosomal protein L23a	9.4	0.7	11.7	6.93E-11	1.58E-08	76	13.5
W5P1X5	phosphohistidine phosphatase 1 (Fragment)	9.4	0.5	12.8	1.35E-11	3.77E-09	62	14.5
W5PVN5	DEAD-box helicase 42	9.4	0	10.6	4.89E-10	8.39E-08	101	11.9
W5PG83	Serine/threonine-protein phosphatase 2A activator (Fragment)	9.3	-1.6	17.6	2.44E-14	2.50E-11	17	19.6
W5PCE0	Phospholipase B-like	-9.3	-5.2	-9.7	2.30E-09	3.25E-07	123	10.7
W5QBE4	Fibrinogen like 2	9.1	-6.3	9.7	2.50E-09	3.42E-07	127	10.2
W5Q1W8	N-acetylglucosamine-1-phosphate transferase gamma subunit	9	-1.4	16	1.53E-13	1.15E-10	23	18.4
W5Q7E2	G protein pathway suppressor 1 (Fragment)	9	-1.1	9	8.18E-09	9.79E-07	145	10
W5Q420	Reticulocalbin 1 (Fragment)	8.7	1.6	10.9	2.63E-10	4.90E-08	93	12.1
W5Q9X5	Mitogen-activated protein kinase kinase 1 (Fragment)	8.6	-1.5	10.6	4.98E-10	8.46E-08	102	12
W5Q702	Keratin 16 (Fragment)	8.4	-7.3	13.1	8.84E-12	2.95E-09	52	14.8
W5Q613	IQ motif containing GTPase activating protein 2	8.1	-1.5	11.8	5.97E-11	1.39E-08	74	14

Disease Models & Mechanisms • Supplementary information

Supplementary table 2d

Protein.ID	Protein.name	logFC	AveExpr	t	P.Value	q.Value	rank	lnOdds
W5PQA0	Beta-microseminoprotein	28.1	-1	13.8	2.90E-12	5.72E-09	8	13.7
W5NWL0	Coiled-coil domain containing 88A	-20.5	-4.8	-7.8	9.95E-08	1.90E-05	91	7.1
W5P240	WAP four-disulfide core domain protein 18-like	-20.5	-2.9	-7.9	8.15E-08	1.61E-05	87	6.9
Q7M371	Plasma proteinase inhibitor (Fragment)	-19.3	3.2	-20.1	1.45E-15	2.52E-11	1	17.7
W5PCE0	Phospholipase B-like (EC 3.1.1.-)	16.7	-5.2	11.4	1.17E-10	9.23E-08	22	10.8
W5PKA9	Coagulation factor V	-15.6	1.9	-8.2	3.88E-08	8.52E-06	79	7.8
W5PYG7	Eukaryotic translation initiation factor 3 subunit L (eIF3l) (Eukaryotic translation initiation factor 3 subunit 6-interacting protein) (Eukaryotic translation initiation factor 3 subunit E-interacting protein)	15.5	-0.4	8.3	3.65E-08	8.23E-06	77	8.1
W5PV90	Eukaryotic translation initiation factor 5B	-15.4	-0.3	-7.6	1.39E-07	2.52E-05	96	6.7
W5Q8R4	DNA helicase (EC 3.6.4.12)	-15.2	-1.6	-7.1	4.75E-07	7.60E-05	108	5.9
W5PTW0	G protein nucleolar 1 (putative)	15	-0.9	10	1.37E-09	6.08E-07	39	9.9
W5QEG5	SPT16 homolog, facilitates chromatin remodeling subunit	15	0.3	6.6	1.31E-06	0.00017	133	5
W5NVG2	EGF containing fibulin like extracellular matrix protein 1	-14.8	1.3	-7.1	4.10E-07	6.72E-05	106	6
W5Q0L1	Eukaryotic translation elongation factor 1 gamma	14.7	0.9	11.6	9.03E-11	8.24E-08	19	12
W5NWP4	DEAD-box helicase 3, X-linked	14.4	-0.2	11.5	1.08E-10	8.89E-08	21	11.9
W5PJG0	Serum amyloid A protein	-14.4	-5.8	-8.7	1.43E-08	3.81E-06	65	8.6
W5QFM1	Histone H2A	-14.3	1	-8.1	4.84E-08	1.01E-05	83	7.7
W5NS65	Histone H1.4	-14.3	-1	-7	5.69E-07	8.81E-05	112	5.7
W5Q7R4	Inhibin beta A chain	-14.3	-0.9	-8.7	1.65E-08	4.34E-06	66	8.5
W5QFP2	Histone cluster 1 H1 family member d	-14.2	0.4	-19.3	3.54E-15	3.07E-11	2	17
W5NYH4	BCL2 associated athanogene 3	14.1	-1.4	7.8	9.06E-08	1.75E-05	90	7.2
W5NR38	RB binding protein 9, serine hydrolase	-14.1	-0.8	-7.1	4.32E-07	7.00E-05	107	6
W5PA79	Splicing factor 3a subunit 1	-14.1	-0.5	-6.7	1.06E-06	0.00014	130	5.3
W5Q831	Destrin, actin depolymerizing factor (Fragment)	14	1.4	9.2	6.05E-09	1.94E-06	54	9.1

W5QCY6	GMP reductase (GMPR) (EC 1.7.1.7) (Guanosine 5'-monophosphate oxidoreductase) (Guanosine monophosphate reductase) (Fragment)	14	0.2	13	9.07E-12	1.21E-08	13	13.5
W5NRI6	Pyridoxal phosphate homeostasis protein (PLP homeostasis protein) (Proline synthase co-transcribed bacterial homolog protein) (Fragment)	13.9	-3.1	6.8	8.13E-07	0.00012	122	5.1
W5QA07	protein HP-25 homolog 1	-13.8	0.5	-6.8	8.89E-07	0.00012	124	5.4
P54230	Cathelicidin-1	-13.7	-0.4	-6.6	1.30E-06	0.00017	132	5
W5P2U4	eukaryotic peptide chain release factor subunit 1	-13.4	-0.7	-6.9	7.10E-07	0.00011	117	5.6
W5Q0C0	Procollagen-lysine,2-oxoglutarate 5-dioxygenase 3	-13.3	0.6	-7	5.91E-07	8.99E-05	114	5.7
W5NTV7	Endothelial differentiation related factor 1 (Fragment)	13.3	-0.3	10.6	4.41E-10	2.18E-07	35	11.1
W5Q7V5	Immunoglobulin superfamily containing leucine rich repeat	13.2	0.1	10.7	3.72E-10	2.02E-07	32	11.3
W5P4S2	Thioredoxin like 1	13.2	-0.2	10.8	3.22E-10	1.86E-07	30	11.4
W5PZ86	Septin 8	13.2	0.8	8.2	4.29E-08	9.30E-06	80	7.8
P21752	P21752 (Bos taurus) Thymosin beta-9	-13.2	-0.1	-15.3	3.85E-13	1.34E-09	5	15.1
W5PXR1	Ectonucleotide pyrophosphatase/phosphodiesterase 1	13.1	-0.7	7	4.92E-07	7.75E-05	110	6
W5PPC2	heterogeneous nuclear ribonucleoprotein D like	13.1	-0.6	10.7	4.24E-10	2.16E-07	34	11.2
W5NQK6	LIM and senescent cell antigen-like-containing domain protein	13.1	0.4	14.1	2.08E-12	5.16E-09	7	14.5
P49929	Cathelin-related peptide SC5	-13.1	-3.6	-7.8	1.04E-07	1.96E-05	92	6.7
W5PKQ5	Microtubule associated protein 1A	-13	-2	-7.7	1.15E-07	2.13E-05	94	7.1
W5QFK2	Microtubule-actin crosslinking factor 1	-13	-1.3	-8.4	2.70E-08	6.60E-06	71	8.2
ENSBTAP000000023055	Hepatocyte growth factor activator preproprotein	-13	0	-6.7	9.30E-07	0.00013	127	5.3
W5PS11	Phosphoserine aminotransferase (Fragment)	-12.9	-1.1	-6.4	2.18E-06	0.00025	149	4.6
W5QED7	ADP-ribosylhydrolase like 2	12.9	0.3	11.3	1.38E-10	9.94E-08	24	11.9

W5PM87	mitogen-activated protein kinase kinase 2	-12.8	-6.7	-6.8	7.71E-07	0.00011	121	5.6
W5P181	Nuclear distribution C, dynein complex regulator	12.8	-1.3	7.3	2.98E-07	5.02E-05	103	6.2
W5PLE0	doublecortin like kinase 1	-12.8	-1.6	-6.4	2.07E-06	0.00024	147	4.7
A2P2G9	VH region (Fragment)	-12.7	-4	-7.5	1.76E-07	3.06E-05	100	6.4
W5PKQ1	Sorbin and SH3 domain containing 3	12.6	-1.1	9.4	3.68E-09	1.25E-06	51	9.7
W5P0N8	Proteasome activator subunit 3	12.5	-0.6	15.7	2.50E-13	1.09E-09	4	15.5
W5NYF4	Peptidoglycan recognition protein 2	-12.5	-2.4	-6.7	9.73E-07	0.00013	129	5.2
W5Q6V5	Dynamin 2	-12.5	-0.7	-6.4	1.97E-06	0.00023	146	4.7
W5Q0A8	Glucosamine-6-phosphate isomerase (EC 3.5.99.6) (Glucosamine-6-phosphate deaminase)	12.5	-0.9	8.4	2.67E-08	6.60E-06	70	8.2
W5Q106	PDZ and LIM domain 4	12.5	-1.8	7.6	1.49E-07	2.67E-05	97	6.8
W5P7W2	Stromal cell derived factor 4 (Fragment)	12.4	-0.9	12.9	1.11E-11	1.35E-08	14	13.3
W5NQ92	elongin B	12.4	0.2	13.2	7.13E-12	1.03E-08	12	13.5
W5Q3G5	Small muscle protein, X-linked	12.2	-1.9	7	5.74E-07	8.82E-05	113	5.8
W5PKP3	Family with sequence similarity 114 member A1	12.1	0.9	6.9	7.26E-07	0.00011	118	5.5
W5QCR6	Microtubule-associated protein	-12.1	-1.7	-9	7.99E-09	2.39E-06	58	9
W5NQM8	Cortactin (Fragment)	-12	0.3	-6.6	1.33E-06	0.00017	134	5.1
B5AZT5	Insulin-like growth factor binding protein-4	-12	-0.4	-7.8	1.05E-07	1.96E-05	93	6.9
W5PHQ4	Nudix hydrolase 2	12	-1	10	1.37E-09	6.08E-07	38	10.2
W5Q649	Breast cancer anti-estrogen resistance 3	11.9	-5.9	11.3	1.37E-10	9.94E-08	23	12
W5PJ59	SH3 domain binding glutamate rich protein	11.9	-1.2	10.7	3.71E-10	2.02E-07	31	11.2
W5NZ17	Cysteine and histidine rich domain containing 1 (Fragment)	-11.9	-0.2	-7	5.04E-07	7.87E-05	111	5.9
W5PG20	Secernin 1 (Fragment)	11.8	-0.5	9.5	3.60E-09	1.25E-06	50	9.5
W5PDD6	Secernin 2 (Frgment)	11.8	-2	11.5	9.63E-11	8.35E-08	20	12.1
W5PSG8	HD domain containing 3	11.8	-1.3	11.2	1.60E-10	1.11E-07	25	11.7
W5NUU7	Cadherin 5	-11.6	1.1	-9.1	7.74E-09	2.36E-06	57	9.1
W5NRM7	Cellular retinoic acid binding protein 1	11.6	2.7	8.4	2.95E-08	7.11E-06	72	7.8
W5PG42	high mobility group box 3	11.6	-1.4	10.9	2.64E-10	1.58E-07	29	11.6

Disease Models & Mechanisms • Supplementary information

W5PES7	Maturin, neural progenitor differentiation regulator homolog (Fragment)	-11.6	-5.6	-9	8.37E-09	2.46E-06	59	9.1
W5QAP3	Target of myb1 membrane trafficking protein (Fragment)	-11.6	-0.6	-10.5	5.77E-10	2.78E-07	36	10.9
W5PEN2	Tumor protein D52	11.5	0.3	13.6	3.92E-12	6.18E-09	11	13.5
W5Q5R5	SAM and HD domain containing deoxynucleoside triphosphate triphosphohydrolase 1	-11.4	-0.7	-8.6	1.77E-08	4.57E-06	67	8.4
W5QDU4	Tetratricopeptide repeat domain 38 (Fragment)	11.3	-2.9	9.6	2.91E-09	1.06E-06	47	9.9
W5PVU5	Proteasome subunit beta type (EC 3.4.25.1)	-11.3	-1.4	-6.5	1.73E-06	0.00021	142	4.8
W5Q5C7	Catenin alpha 1	11.3	-1.2	8	6.60E-08	1.35E-05	85	7.5
W5Q9C4	Nitrilase family member 2	11.2	-1.1	18.5	8.63E-15	4.99E-11	3	16.8
W5Q3D2	Sorting nexin 12	11.2	0.6	11	2.29E-10	1.42E-07	28	11.1
W5PSB5	GDP-mannose pyrophosphorylase B	11.2	-1.4	12.9	1.17E-11	1.35E-08	15	12.3
B6E3I8	Enolase 1 (Fragment)	-11.2	0.2	-13.7	3.30E-12	5.72E-09	10	14.1
W5PMG4	PYM homolog 1, exon junction complex associated factor	11.1	-1.4	9.6	2.95E-09	1.06E-06	48	9.6
W5Q4B7	Rap1 GTPase-GDP dissociation stimulator 1	-11.1	-2.8	-6.6	1.40E-06	0.00018	137	4.9
W5PV54	Carboxypeptidase N subunit 1	-11.1	-1.2	-6.5	1.51E-06	0.00019	141	4.9
W5NPC7	Acyl-CoA binding domain containing 3 (Fragment)	11	0.9	10.4	7.00E-10	3.28E-07	37	10.4
W5PY64	H1 histone family member X (Fragment)	-11	-1.2	-6.8	7.66E-07	0.00011	120	5.5
W5PXZ3	alpha-fetoprotein-like	-11	-1.3	-6.8	8.53E-07	0.00012	123	5.3
W5PHU8	aminoacyl tRNA synthase complex-interacting multifunctional protein 1	-10.9	-0.9	-6.5	1.48E-06	0.00018	139	4.9
W5PI22	canopy FGF signaling regulator 2	10.9	1.9	9	9.22E-09	2.67E-06	60	8.7
W5PLS7	Growth factor receptor bound protein 2	-10.9	-0.4	-9.9	1.60E-09	6.65E-07	41	10.2
Q8HXS2	Ribosomal protein L19 (Fragment)	-10.8	1.6	-6.8	9.19E-07	0.00013	125	5.2
ENSBTAP0 00000241 46	Alpha-2-macroglobulin isoform X4	-10.8	-5.6	-6.6	1.40E-06	0.00018	136	4.4
C9E8M7	Cytochrome b5	-10.7	-0.3	-6.6	1.19E-06	0.00016	131	4.9

B3GS77	Insulin-like growth factor binding protein 5	-10.7	2	-8.9	9.63E-09	2.74E-06	61	8.4
W5NS79	bifunctional 3'-phosphoadenosine 5'-phosphosulfate synthase 2-like	10.6	-2	8.9	1.06E-08	2.95E-06	62	8.4
W5QFC2	Four and a half LIM domains 3	-10.6	-1.9	-7.9	7.19E-08	1.45E-05	86	7.4
W5NUW3	Amine oxidase	-10.6	-5.5	-6.4	2.08E-06	0.00024	148	4.4
W5PIF5	myristoylated alanine rich protein kinase C substrate	-10.6	-0.5	-6.6	1.35E-06	0.00017	135	5
W5QGL6	p21 (RAC1) activated kinase 2	-10.5	-0.4	-8.3	3.46E-08	7.89E-06	76	8
W5PCD9	Echinoderm microtubule associated protein like 4	10.5	-6.2	6.4	1.86E-06	0.00022	144	4.7
W5NSN2	Collagen type XVI alpha 1 chain	-10.5	1.4	-6.4	1.84E-06	0.00022	143	4.7
W5PV21	Translin	-10.5	-1.9	-9.3	5.09E-09	1.70E-06	52	9.3
W5PUT8	Actin-related protein 2/3 complex subunit 5	-10.5	-1.4	-6.4	1.95E-06	0.00023	145	4.6
W5Q8X3	Metallo-beta-lactamase domain containing 1 (Fragment)	10.4	-3	13.8	3.24E-12	5.72E-09	9	13.6
W5PVZ9	Dystroglycan 1	-10.4	0.7	-6.7	9.29E-07	0.00013	126	5.1
W5P1I7	Ubiquitin conjugating enzyme E2 M	-10.4	-0.8	-9.1	7.56E-09	2.34E-06	56	9.1
W5NSD5	RAP1B, member of RAS oncogene family	-10.4	-0.6	-6.3	2.38E-06	0.00027	150	4.5
W5PHQ0	Myeloperoxidase (Fragment)	-10.3	-5	-9.9	1.65E-09	6.65E-07	43	9.9
W5PKS8	DEK proto-oncogene	-10.3	-0.5	-9.2	5.55E-09	1.82E-06	53	9.3
W5PVN5	DEAD-box helicase 42	-10.3	0	-8.3	3.27E-08	7.67E-06	74	7.5
W5PPZ9	guanidinoacetate N-methyltransferase (Fragment)	-10.2	-1.8	-8.2	4.52E-08	9.56E-06	82	7.7
W5PP53	Cold inducible RNA binding protein	10.2	-1.2	7.9	8.16E-08	1.61E-05	88	7.3
W5PME6	Zinc finger CCCH-type containing, antiviral 1 like	10.1	-0.2	6.9	6.78E-07	0.0001	116	5.6
W5P1T4	Serine/threonine-protein phosphatase	10.1	-6.2	8.3	3.40E-08	7.87E-06	75	7.8
W5QHV6	Sorting nexin 1	10.1	0.8	8.6	1.96E-08	5.00E-06	68	8
W5PZT2	Angiopoietin like 6	-10	-6	-8.8	1.18E-08	3.19E-06	64	8.5
C5IWV3	Protein disulfide isomerase family A member 5	-10	-0.8	-9.9	1.48E-09	6.41E-07	40	10.1

Disease Models & Mechanisms • Supplementary information

W5PPQ6	SEC13 homolog, nuclear pore and COPII coat complex component	10	0.1	11.2	1.68E-10	1.12E-07	26	11.4
W5NUZ1	Dynein cytoplasmic 1 intermediate chain 2	9.9	-0.9	14.5	1.21E-12	3.51E-09	6	14.2
W5NY25	Eukaryotic translation initiation factor 3 subunit C	-9.9	0.1	-6.7	9.67E-07	0.00013	128	5.3
W5PZ50	TBC1 domain family member 15 (Fragment)	-9.9	-0.3	-7.9	8.27E-08	1.61E-05	89	7
W5P6V4	Golgi glycoprotein 1	9.9	-1.2	8.9	1.13E-08	3.11E-06	63	8.3
W5P7G7	Proteasome subunit beta type (EC 3.4.25.1)	-9.8	-1	-12.6	1.85E-11	1.89E-08	17	13
W5PTM9	ubiquitin conjugating enzyme E2 V2 (Fragment)	-9.8	-0.8	-7.6	1.54E-07	2.73E-05	98	6.7
W5PR73	Coronin (Fragment)	9.6	0.2	7.3	2.87E-07	4.89E-05	102	6.1
W5PK29	Striatin	9.5	-5.1	9.7	2.28E-09	8.81E-07	45	9
W5P6P2	ribosomal protein L7	-9.5	-1.2	-7.2	3.69E-07	6.15E-05	104	5.7
W5Q7E2	G protein pathway suppressor 1 (Fragment)	-9.4	-1.1	-6.6	1.42E-06	0.00018	138	4.9
W5P1X5	phosphohistidine phosphatase 1 (Fragment)	-9.4	0.5	-9.6	2.69E-09	1.01E-06	46	9.4
W5P8I5	elongin C	9.3	0.9	7	4.78E-07	7.60E-05	109	5.7
W5PG83	Serine/threonine-protein phosphatase 2A activator (EC 5.2.1.8) (Phosphotyrosyl phosphatase activator) (Fragment)	-9.3	-1.6	-12.4	2.36E-11	2.28E-08	18	12.9
W5PQQ6	NECAP endocytosis associated 2	-9.2	-7	-8.1	4.99E-08	1.03E-05	84	7.5
W5PDL7	Polypeptide N-acetylgalactosaminyltransferase (EC 2.4.1.-) (Protein-UDP acetylgalactosaminyltransferase)	9.2	-5.8	11.1	1.90E-10	1.22E-07	27	11.1
W5QI48	Endosulfine alpha	-9	-0.3	-12.6	1.67E-11	1.81E-08	16	13.1
W5QE39	Ribosomal protein L23a	-9	0.7	-8.3	3.71E-08	8.26E-06	78	7.6
W5QBE4	Fibrinogen like 2	-9	-6.3	-7.5	1.74E-07	3.05E-05	99	6.2
W5Q613	IQ motif containing GTPase activating protein 2	-8.8	-1.5	-9.1	6.87E-09	2.17E-06	55	9.1
W5PWZ2	Galectin (Fragment)	-8.6	-5.5	-8.5	2.52E-08	6.33E-06	69	7.8
W5Q1W8	N-acetylglucosamine-1-phosphate transferase gamma subunit	-8.5	-1.4	-10.7	3.85E-10	2.03E-07	33	11.1
O62804	Transcription factor GABP alpha subunit (Fragment)	8.5	-6.5	6.8	7.50E-07	0.00011	119	5.2
W5Q420	Reticulocalbin 1 (Fragment)	-8.5	1.6	-8.2	4.46E-08	9.55E-06	81	7.3

Disease Models & Mechanisms • Supplementary information

W5Q9X5	Mitogen-activated protein kinase kinase 1 (Fragment)	-8.2	-1.5	-7.5	1.88E-07	3.24E-05	101	6.4
W5Q702	Keratin 16 (Fragment)	-8.2	-7.3	-9.7	2.24E-09	8.81E-07	44	9.5
W5Q149	Oxidation resistance 1	-7.9	-6.1	-9.9	1.61E-09	6.65E-07	42	9.8
W5PG09	Phosphoglycerate kinase	-7.8	-6.5	-6.5	1.49E-06	0.00018	140	4.7
W5PTH6	Amidophosphoribosyltransferase	7.8	-6.8	7.1	3.87E-07	6.39E-05	105	5.7
W5PS78	opioid growth factor receptor (Fragment)	-7.3	-6.8	-6.9	6.31E-07	9.53E-05	115	5.5
W5QD75	G protein subunit alpha i3 (Fragment)	-6.7	-6.3	-9.6	2.99E-09	1.06E-06	49	9.4
W5Q9P0	Catenin beta like 1	-6.3	-6.5	-8.3	3.20E-08	7.61E-06	73	7.7
W5Q2B6	Epiphycan	6.1	7.1	7.7	1.26E-07	2.30E-05	95	6.7
